# Interplay between transglutaminases and heparan sulphate in progressive renal scarring

**DOI:** 10.1038/srep31343

**Published:** 2016-10-03

**Authors:** Izhar Burhan, Giulia Furini, Hugues Lortat-Jacob, Adeola G. Atobatele, Alessandra Scarpellini, Nina Schroeder, John Atkinson, Mabrouka Maamra, Faith H. Nutter, Philip Watson, Manlio Vinciguerra, Timothy S. Johnson, Elisabetta A. M. Verderio

**Affiliations:** 1 Nottingham Trent University, School of Science and Technology, Nottingham, NG11 8NS, United Kingdom; 2 Institut de Biologie Structurale, UMR 5075, Univ. Grenoble Alpes, CNRS, CEA, Grenoble, F-38027, France; 3 University of Sheffield, Academic Nephrology Unit, Medical School, Sheffield, S10 2RZ, United Kingdom

## Abstract

Transglutaminase-2 (TG2) is a new anti-fibrotic target for chronic kidney disease, for its role in altering the extracellular homeostatic balance leading to excessive build-up of matrix in kidney. However, there is no confirmation that TG2 is the only transglutaminase involved, neither there are strategies to control its action specifically over that of the conserved family-members. In this study, we have profiled transglutaminase isozymes in the rat subtotal nephrectomy (SNx) model of progressive renal scarring. All transglutaminases increased post-SNx peaking at loss of renal function but TG2 was the predominant enzyme. Upon SNx, extracellular TG2 deposited in the tubulointerstitium and peri-glomerulus via binding to heparan sulphate (HS) chains of proteoglycans and co-associated with syndecan-4. Extracellular TG2 was sufficient to activate transforming growth factor-β1 in tubular epithelial cells, and this process occurred in a HS-dependent way, in keeping with TG2-affinity for HS. Analysis of heparin binding of the main transglutaminases revealed that although the interaction between TG1 and HS is strong, the conformational heparin binding site of TG2 is not conserved, suggesting that TG2 has a unique interaction with HS within the family. Our data provides a rationale for a novel anti-fibrotic strategy specifically targeting the conformation-dependent TG2-epitope interacting with HS.

Chronic kidney disease (CKD) such as glomerulonephritis and diabetic nephropathy instigates a progressive remodelling process leading to renal scarring, fibrosis and, ultimately, kidney failure. This is characterised by excessive accumulation of extracellular matrix proteins (ECM), fibroblast proliferation and tubular atrophy^1^. Abundant fibrillary collagens (type I and II) and capillary basement membrane, consisting of collagen IV, V and other proteins like fibronectin, laminin and proteoglycans^2^, deposit in the tubular interstitial space between tubules and peritubular capillaries, impairing the waste and nutrients exchange-function of tubules. As the disease progresses, further matrix expansion leads to loss of nephrons and capillaries, ultimately leading to loss of kidney function. Since accumulation of interstitial ECM is associated with a decline in renal function, inhibitors of ECM accumulation are anti-fibrotic in experimental models of kidney fibrosis^1^.

There is now considerable data indicating that the wound response enzyme family of transglutaminases (TG) are integral in the process of ECM expansion. Transglutaminases catalyse the post-translational modification of proteins predominantly via cross-linking the γ-carboxamide group of a peptide-bound Gln residue and either the ε-amino group of a peptide-bound Lys residue on adjacent polypeptides or a primary amino group of polyamine^3^. Protein cross-linking depends on Ca^2+^ binding and GTP dissociation, conditions which are favoured in the extracellular environment or following cell injury and loss of Ca^2+^ homeostasis. Several transglutaminases have been characterised with distinct genes, structures and physiological functions^3^. Examples are factor XIIIa (FXIIIa), which stabilises fibrin during blood clotting, and TG1, which has a role in skin barrier formation. The most widespread member of this family, transglutaminase-2 (TG2), has a clear fibrogenic role contributing to the stabilisation and accumulation of the ECM consequent to lung, liver, heart and kidney fibrosis^4^,^5^,^6^,^7^,^8^,^9^,^10^. Extracellular TG2 has a number of substrates in the ECM including fibronectin and collagen XVIII/endostatin, specialised glycoproteins with a core protein linked to heparan sulphate side chains^11^,^12^. Some substrates of TG2, like fibronectin, are in common with other TG members such as FXIIIa^13^. TG2 activity typically stabilises and increases fibronectin and collagen deposition^3^,^14^, as well as the activity of transforming growth factor beta-1 (TGF-β1)^15^,^16^, a central mediator of the over-proliferation of fibroblasts and myofibroblasts leading to organ fibrosis^17^. Several studies have clearly shown that modulation of extracellular TG-mediated transamidation significantly affects kidney scarring^8^,^9^,^10^,^18^, and that chemical inhibition of all TG family members ameliorates tubulointerstitial fibrosis^18^,^19^,^20^. TG2 can be ascribed a role in changing renal ECM homeostasis^14^, however there is no confirmation that TG2 is the key player, neither there are strategies to control/prevent the fibrogenic action of extracellular TG2 specifically in human disease.

New observations, both *in vitro* and *in vivo,* indicate that the biological role of TG2 may be modulated by its interaction with heparan sulphate proteoglycans (HSPG), key components of the tubular basement membrane^21^. We have demonstrated that the heparin binding site of TG2 comprises clusters of basic amino acids that are brought together on the folded nucleotide-bound conformation^22^, and that this is critical in cell-ECM interactions^23^. Cell surface TG2 interacts with matrix HSPG which affect its extracellular trafficking^24^, but once released and deposited in the matrix, TG2 interacts back with cell surface HSPG inducing outside-in signalling^25^,^26^. We have recently reported that knock-out (KO) of the HSPG receptor syndecan-4 (Sdc4) ameliorates fibrosis in the murine unilateral ureteric obstruction (UUO) and aristolochic acid nephrotoxicity (AAN) models, and that this is connected with a lower level of extracellular TG2 protein and activity^27^.

Despite advances on the role of TG2 in kidney scarring, the expression of other TG isoenzymes, also capable of protein transamidation but individual in expression pattern, subcellular location and regulation, has never been analysed and it remains a possibility that other TG (TG1, TG3, TG5-7, Factor XIIIa)^28^ could contribute to the disease process. Moreover, TG2 itself comprises a number of transcript variants, corresponding to predicted truncated TG2 proteins which are intrinsically more active in protein transamidation as they lack a critical C-terminal GTP binding site^29^. Therefore, understanding whether TG2 is the main form implicated in fibrosis progression, whether its interaction with HS is unique and its significance in fibrosis development is a key goal for the development of new treatment strategies that target TG2.

To address this need, we have measured the expression of TG family members in the 5/6th subtotal nephrectomy (SNx) model of renal scarring, which is regarded as a useful reproduction of chronic kidney scarring, comparable to that observed in human disease. We showed the predominance of TG2 over other TG family members in the disease process, its co-association with Sdc4/HS in the tubulointerstitium, and a role for HS in modulating TGF-β1 activation via extracellular recruitment of TG2. This study broadens our understanding of the interplay between TG2 and HS/Sdc4 in TGF-β1 activation and fibrosis.

## Results

### Expression profiling of TG and syndecan family members in the SNx model of progressive kidney fibrosis

#### Transglutaminases

**Figure 1:**
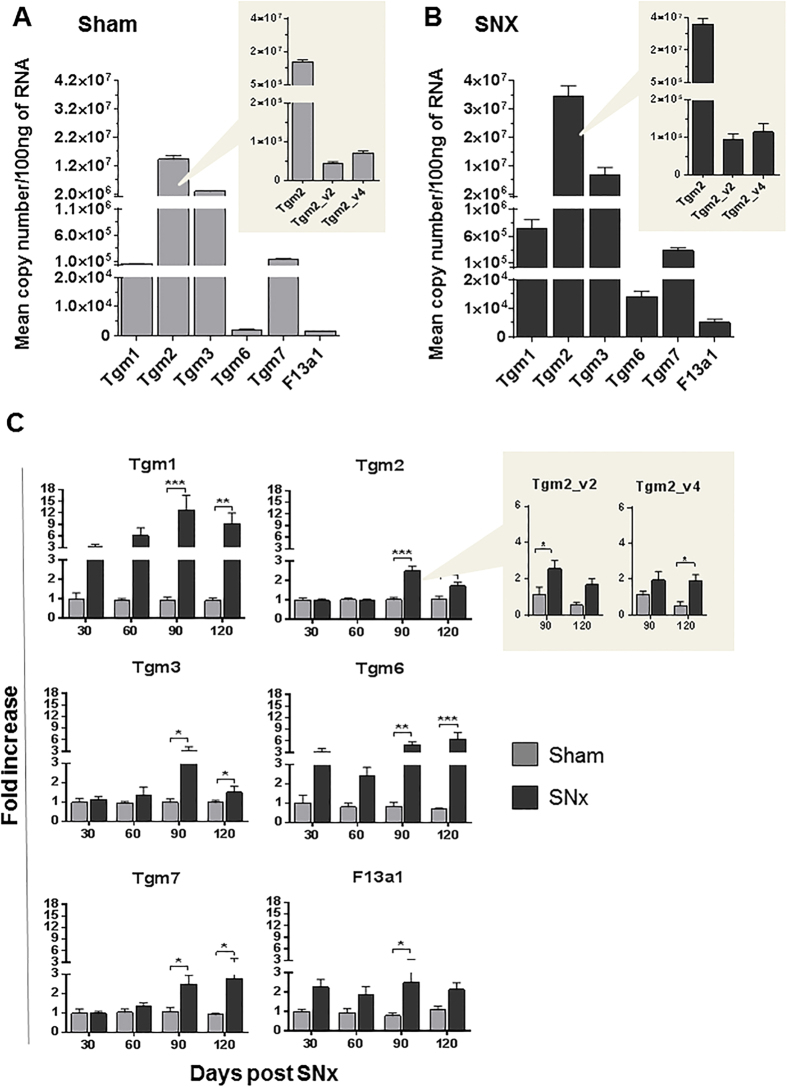
Expression profiling of TG family members in normal rat kidney and in kidney post-SNx. The mRNA expression level of six TG and two *Tgm2* alternative transcripts was quantified in total RNA of rat kidneys at 30, 60, 90 and 120 days post-SNx and control kidneys of sham-operated rats. Quantifications were carried out by qRT-PCR, using isoform-specific oligonucleotide-primers, reported in Suppl. Table 1. (**A**,**B**) Absolute quantifications of expression of TG family members and *Tgm2* isoforms (inset) were performed in sham operated kidneys (**A**) and kidneys at 90 days post-SNx (**B**). Data are mean copy number/100 ng RNA ± SEM. At least four kidneys were tested per time point. Data were normalised to the expression of housekeeping gene cyclophilin A (*Ppia*), but they were not significantly altered by normalisation, suggesting good experimental reproducibility. (**C**) The fold changes in mRNA expression were measured during the progression of renal scarring, at days 30, 60, 90 and 120 post-SNx. Values are mean fold changes in canonical TG genes expression (*Tgm*) and alternative *Tgm2* gene transcripts (inset) post-SNx, relative to the corresponding untreated control (sham-operated rats). Data were normalised to *Ppia* values and expressed relative to expression at 30 days in the sham-operated kidney (equalised to 1).

We quantified the expression level of TG genes in rat kidneys subjected to the 5/6^th^ subtotal nephrectomy (SNx) model of progressive renal scarring. In sham-operated control kidneys, *Tgm2* was found to be predominantly expressed, when compared with the other transglutaminase members, as measured by qRT-PCR (Fig. 1A). *Tgm5* was the only not detectable member in rat kidney. Using oligonucleotides specific for the rat *Tgm2* splice forms *Tgm2_v2* 30,31,32 and *Tgm2_v4* 31,33 we found expression of these variants although they were only less than 1% of the total *Tgm2* transcript (Fig. 1A, inset). Transcript levels of all TG family members steadily increased post-SNx compared to the corresponding sham-operated controls (Fig. 1C), with maximum expression levels by day 90 (corresponding to advanced fibrosis) or day 120 post-SNx (corresponding to end-stage kidney failure). The *Tgm2* rat variants (*Tgm2_v2 and Tgm2_v4*), were also found to significantly increase post-SNx (Fig. 1C, inset). Neither glyceraldehyde 3-phosphate dehydrogenase (*Gapdh*) nor cyclophilin A (*Ppia*) houseekeeping gene transcripts significantly changed at all time points compared to control kidneys (Suppl. Fig. 1). These data show that the expression of all identified TG family members is increased in renal scarring at some stage. However, at 90 days post-SNx, absolute values of *Tgm2* expression (Fig. 1B) still exceeded that of all the other analysed TGs, and *Tgm2* was approximately 4 times greater than the next most expressed TG form (*Tgm3*). The *Tgm2* rat variants remained less than 1% the level of the total *Tgm2* transcript (Fig. 1B, inset).

**Figure 2:**
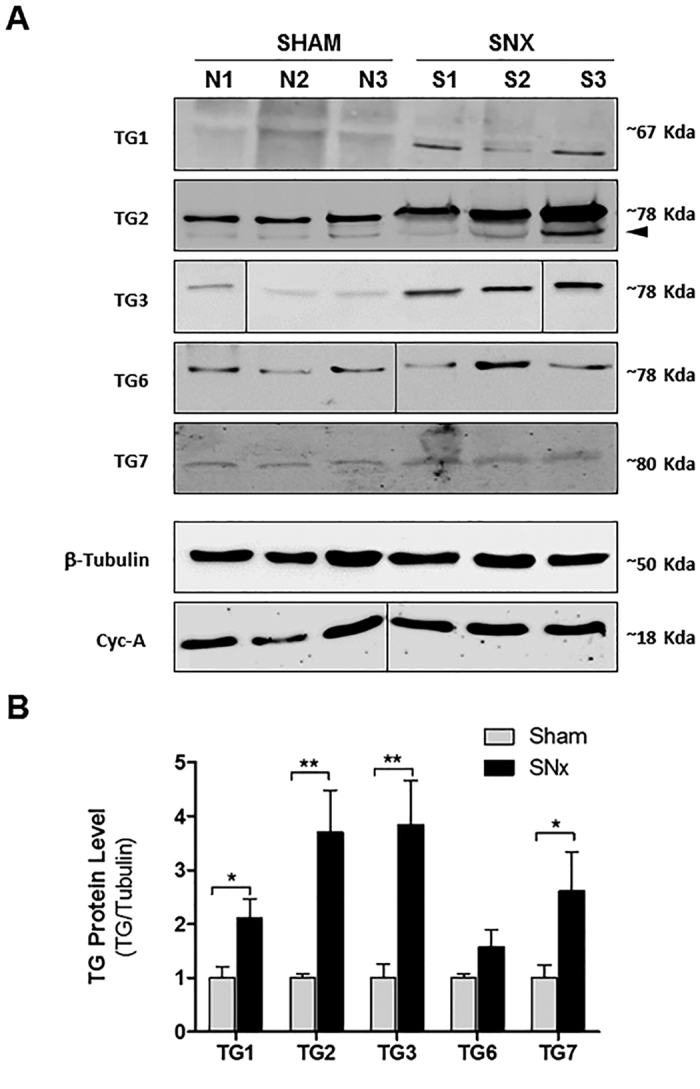
TG protein family expression post-SNx. Kidney homogenates were obtained from snap-frozen kidney portions of three randomly selected rats post-SNx and sham-operated, as described in the Methods. (**A**) Proteins (corresponding to 100 μg of genomic DNA) were separated by reducing SDS-PAGE (10% w/v) and immunoblotted for detection of TG1, TG2, TG2, TG6, TG7 using specific antibodies (stated in Supplementary Information). Cyclophilin A (Cyc-A) and β-tubulin were also immunodetected to control the loading. In the TG2 blot the arrow points at a band with size consistent with the predicted mass of rat TGM2_v4 (73 kDa). (**B**) TG protein levels were evaluated by densitometric analysis and expressed as mean ± SEM of TG2/β-tubulin.

Differences in TG protein production in kidneys undergoing SNx was shown by western blotting of sham-operated and fibrotic rat kidney lysates post-SNx, using isoform-specific antibodies (Fig. 2A). Densitometric analysis showed that all the TG isoforms increased post-SNx at a stage of “advanced fibrosis”, with a trend to rise also for TG6 (Fig. 2B), and confirmed that TG2 is the most expressed family member post-SNx. FXIIIa expression was negligible and it was omitted from the figure. Induction of TG protein expressions post-SNx were consistent with induction of gene expression measured by qPCR (Fig. 1C). However, the fold changes at transcript level did not always result in the same fold changes at protein level and this may be due to different post-transcriptional mechanisms that are not uncommon for TG^34^. The TG2 rat protein variants resulting from translation of rat *Tgm2_v2* and rat *Tgm2_v4* transcripts (predicted molecular mass of 78 kDa and 73 kDa respectively (Suppl. Table 1)) could not be identified conclusively as close in molecular mass to the main TG2 variant (77 kDa), although the immunoblot revealed a lower compatible band below the main TG2 variant (Fig. 2A, arrow).

#### Syndecans

**Figure 3:**
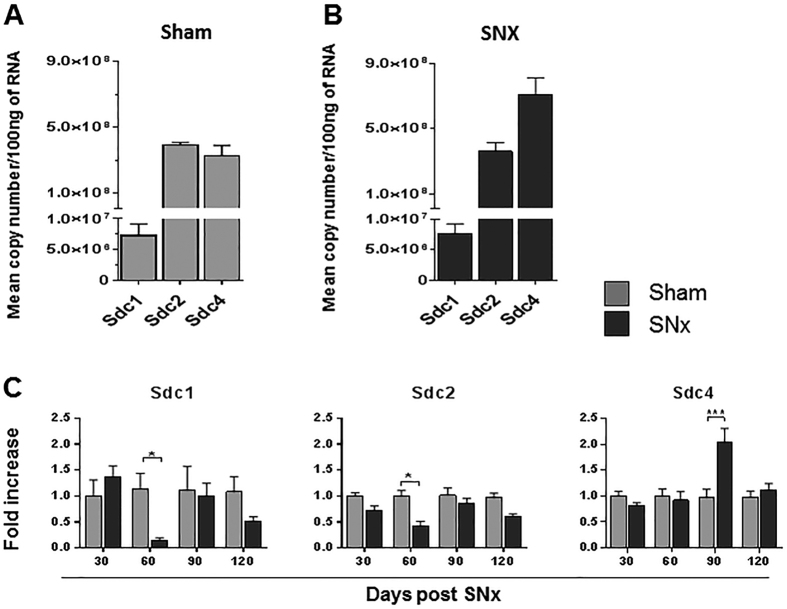
Expression profiling of syndecan family members in normal rat kidney and in kidney post-SNx. The mRNA expression level of three syndecan family members was quantified as described in legend to Fig. 1. (**A**,**B**) Absolute quantifications of expression of *Sdc* in sham operated kidneys (**A**) and kidneys at 90 days post-SNx (**B)**. (**C**) The fold changes in mRNA expression were measured during the progression of renal scarring, at days 30, 60, 90 and 120 post-SNx. Values are mean fold changes of *Sdc* genes expression relative to the corresponding untreated control. Data were normalised to *Ppia* values and expressed relatives to expression at 30 days in the sham-operated kidney (equalised to 1).

**Figure 4:**
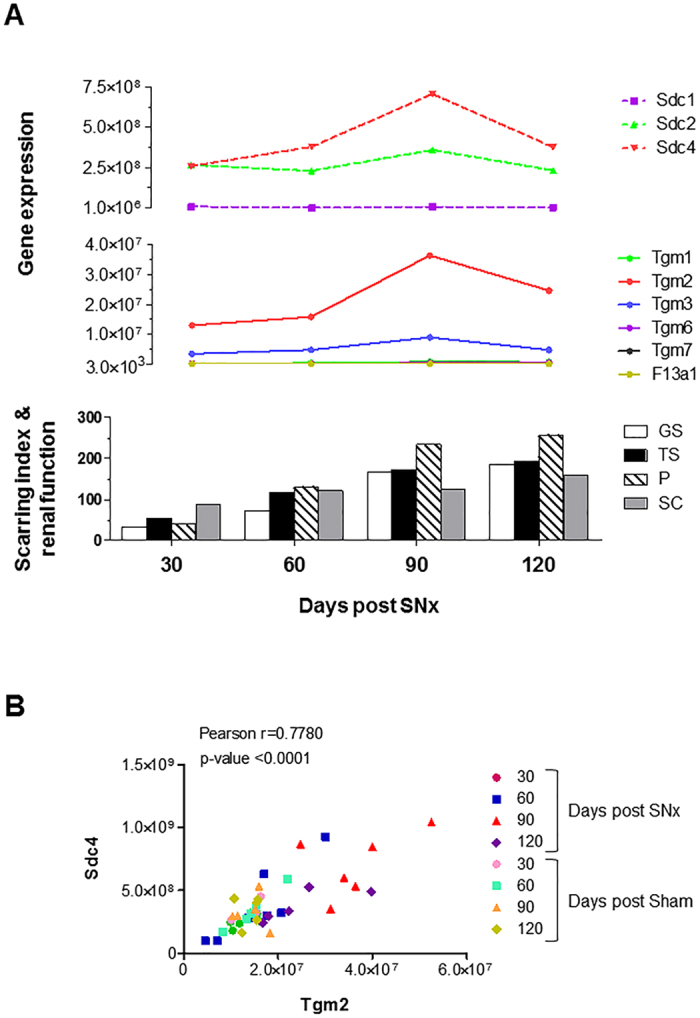
Correlation of TG2 and Sdc4 gene expression with progressive renal scarring in the SNx model of kidney fibrosis. (**A**) Expression profile of *Tgm* and *Sdc* genes at increasing indices of peri-glomerular scarring (GS), tubular-interstitial scarring (TS), proteinuria (P) and serum creatinine (SC) post-SNx, as reported^35^. The scarring index and serum creatinine values are expressed as 100 times the original values. (**B**) The mean copy numbers for *Sdc4* and *Tgm2* at days 30, 60, 90 and 120 post-SNx and corresponding control was utilised to calculate the Pearson correlation coefficients (n = 24 for SNx and n = 19 for controls).

To determine whether syndecan family members correlated with progressive renal scarring, we profiled the expression level of syndecan genes post-SNx. Although *Sdc4* and *Sdc2* showed similar levels in control kidney, and were more expressed than *Sdc1* (Fig. 3A), *Sdc4* was the highest expressed syndecan post-SNx (Fig. 3B) and the only member induced at 90 days post-SNx (Fig. 3C). Intriguingly, both *Sdc1* and *Sdc2* transcripts dropped at the beginning of the fibrotic response to then recover and reach control levels of expression (Fig. 3C), suggesting that their transitory switch-off may be part of the cell transformation events causing fibrosis progression. As shown in Fig. 4, when the expression profiles of TG and syndecans were superimposed with parameters of renal scarring progression (peri-glomerular and tubular) and renal insufficiency (proteinuria and serum creatinine) of the rat kidneys post-SNx^35^, the expression of *Tgm2* and *Sdc4* were predominant, paralleled disease development (Fig. 4A) and significantly correlated in the SNx model (Fig. 4B). As both *Tgm2* and *Sdc4* peaked at “advanced fibrosis” (90 days post-SNx) (Fig. 4), this suggests that the dynamic of TG2-Sdc4 interaction intensifies mid-way through the scarring progression when the process becomes irreversible.

### Heparin binding properties of TG members

**Figure 5:**
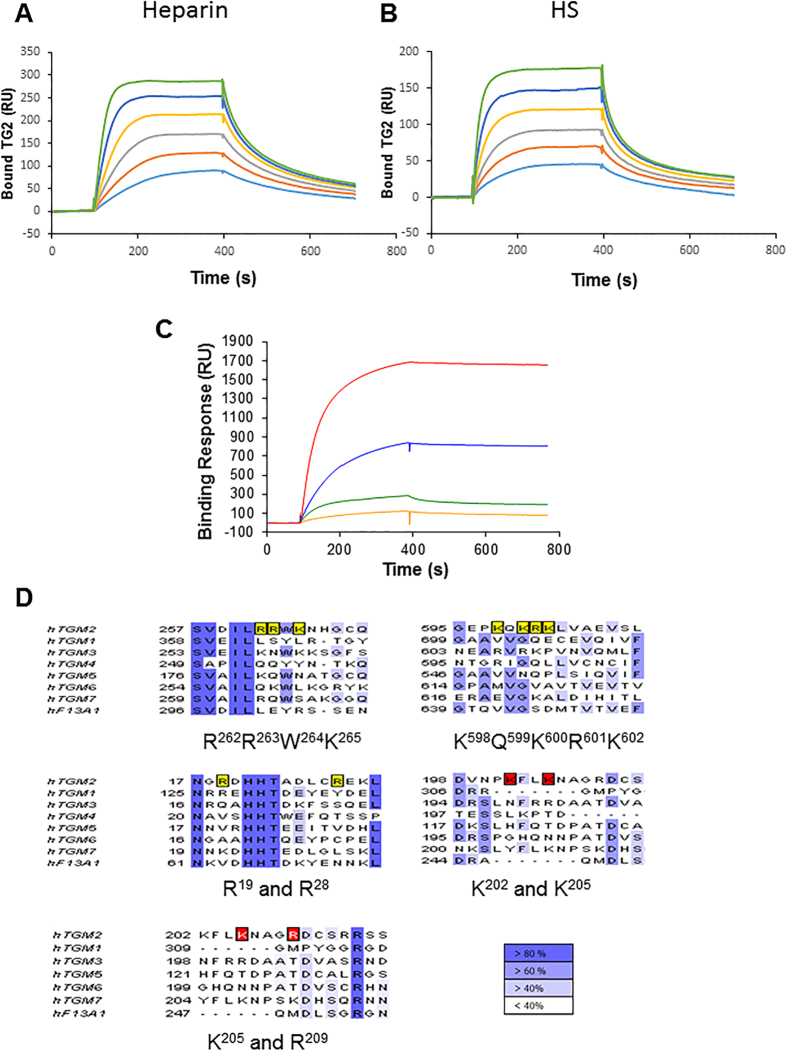
Binding of TG family members to immobilized HS. Recombinant human TGs were injected over a heparin-activated surface at a flow rate of 20 μl/min for 5 min and the response in resonance units (RU) was recorded as a function of time. (**A,B**) Binding of TG2 at 66, 100, 150, 222, 333 and 500 nM to heparin (50 RU immobilized) (**A**) and to HS (45 RU immobilized) (**B**). (**C**) Comparison of the heparin binding activity of TG1 (red), TG2 (blue), TG3 (green) and FXIIIa (orange), all at 300 nM. (**D**) The catalytically active human TGs were aligned using ClustalW and viewed in Jalview. Boxed residues in yellow and red correspond to amino acid residues important for heparin binding within TG2 according to Lortat-Jacob *et al*.^22^ and Wang *et al*.^39^. Different shades of blue define the percentage of residues in each column that agree with the consensus sequence.

The heparin binding properties of TG family members mostly expressed in the fibrotic kidneys, TG2, TG1 and TG3, and of FXIIIa, since present in plasma and secreted in the ECM^36^, were investigated by a solid phase assay. Surface plasmon resonance (SPR) spectroscopy was used to measure changes in the refractive index caused by the interaction that occurred when the various TG were flowed across a biotinylated heparin surface, captured on top of a streptavidin-coated sensorchip. Data (Fig. 5) indicated a relatively strong binding between TG2 and heparin (Fig. 5A) and between TG2 and purified HS, a more physiological ligand (Fig. 5B). The value at equilibrium was lower for HS than for heparin, and this was expected since unlike heparin, which is sulphated all along the chain, HS has only short segments of its sequence sulphated. There was a moderate binding between TG3 and heparin and only a weak interaction of FXIIIa with heparin (Fig. 5C). Surprisingly, TG1 displayed a very strong interaction with heparin (Fig. 5C). However, the tendency of TG1 to oligomerise (“Crystal structure of the human transglutaminase 1 beta-barrel domain” by Vollmar *et al*. (PDB 2XZZ)) is likely to affect the SPR detection, which is directly sensitive to the mass of material binding to the sensor surface.

Sequence alignments performed in Jalview^37^ (Fig. 5D), showed that the previously described TG2 heparin binding site, consisting of two positive clusters comprising 3 (RRWK_262–265_) and 4 (KQKRK _598–602_) basic residues, and the contributing amino acids Arg_19_ and Arg_28_^22^, had no apparent homologous sequences in TG1. Only the basic residue Arg_19_ was conserved in TG1 and accessible in the predicted 3D structure^38^. A further proposed heparin binding site in TG2 across amino acids 202–215 39,40 also appeared not conserved in TG1. These data suggest that the TG1 interaction with heparin/HS may occur via a distinct binding site. TG3 displayed two positive clusters in sequences analogues to those in TG2 (Fig. 5D), consisting of a weaker dibasic residue cluster, KNWK _259–262_, and a tribasic residues cluster RVRK_606–609_. Indeed these could serve as a less strong heparin binding site, given that TG3 has a folded structure similar to that of TG2 41. FXIIIa had no obvious homology with the heparin binding sequences identified in TG2 (Fig. 5D) although its monomer structure is close to that of TG2 42; also TG6 and TG7 did not show obvious analogy with the TG2 positive clusters (Fig. 5D). Therefore, it appears that TG2 has a unique heparin binding site, which has some potential weaker analogy only in TG3.

### The extracellular deposition of TG2 in scarred kidney depends on HS proteoglycans

#### Wash-out of matrix TG2 by HS digestion

**Figure 6:**
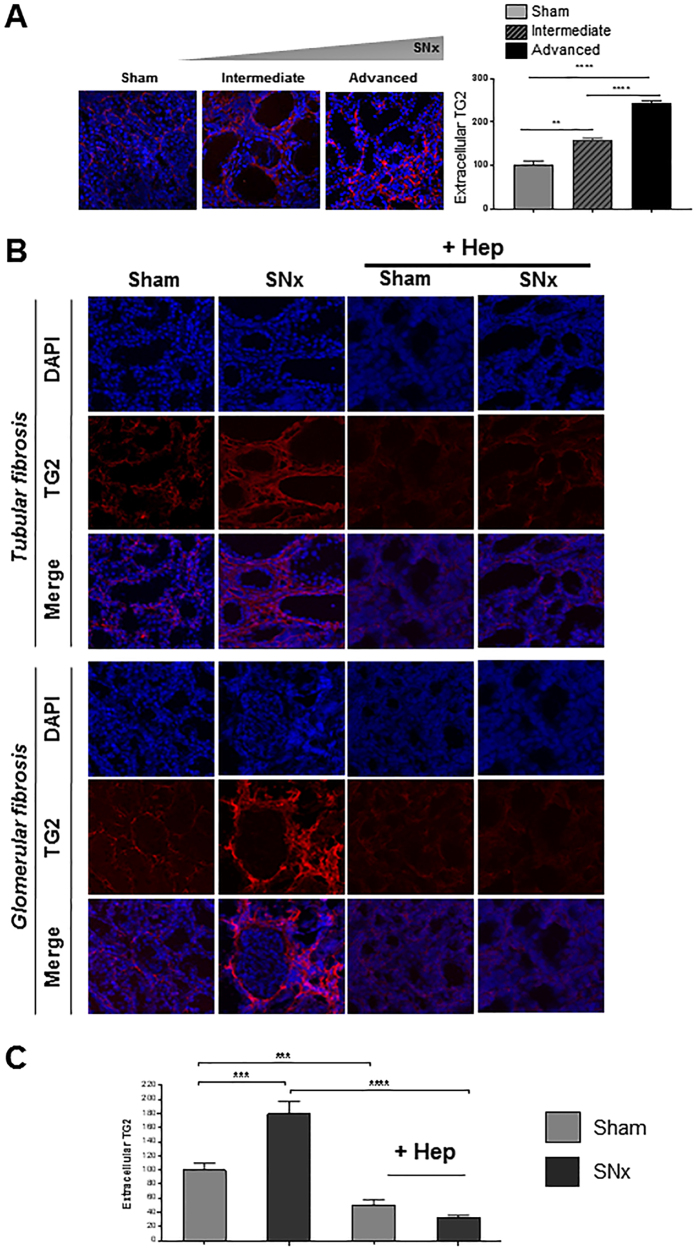
Increase in extracellular TG2 deposition in SNx kidneys and TG2 “wash-out” by specific HS cleavage. Unfixed kidney cryosections were immunolabeled using mouse monoclonal anti-TG2 antibody IA12, followed by goat anti-mouse IgG-DyLight 594 antibody and nuclear staining with DAPI. (**A**) Representative confocal images of TG2 and DAPI-stained sections for sham-operated and post-SNx kidneys at intermediate and advanced fibrosis and quantification of TG2 intensity by image analysis (multiple random fields). (**B**) Two advanced fibrotic lesions are displayed showing extracellular TG2 predominantly localised in the tubulointerstitial space and in the peri-glomerular area around Bowman’s. Where indicated, sections were pre-treated with 50 mU/ml protease-free heparitinase I (+Hep) for 2 hours at 37 °C followed by TG2 staining. (**C**) Extracellular TG2 was quantified by ImageJ intensity analysis in four kidneys per treatment (3 sections per kidney; 4–7 non overlapping images per section). Magnification, 63X.

Immunofluorescence staining of extracellular TG2 was visualised in unfixed cryostat sections of kidneys of SNx- or sham-operated rats at intermediate and advanced fibrosis (Fig. 6A). There was a gradual loss of architecture, with flattened and expanded tubules post-SNx compared to control, and TG2 progressively deposited in the extracellular space as the disease model progressed (Fig. 6A). In advanced fibrosis, TG2 accumulated in the tubular interstitium and in the peri-glomerular area around the Bowman’s capsule in the fibrotic kidneys, consistent with gene expression evaluations (Fig. 6B). Selective cleavage of HS chains by heparitinase I (Fig. 6B, +Hep) significantly reduced the level of extracellular TG2 below control levels, as confirmed by image analysis (Fig. 6C). This finding is consistent with previous observations in the mouse AAN model^27^ and suggests that HS plays a critical role in trapping TG2 in the extracellular space of rat kidneys post-SNx.

#### Co-association of TG2 and Sdc4

**Figure 7:**
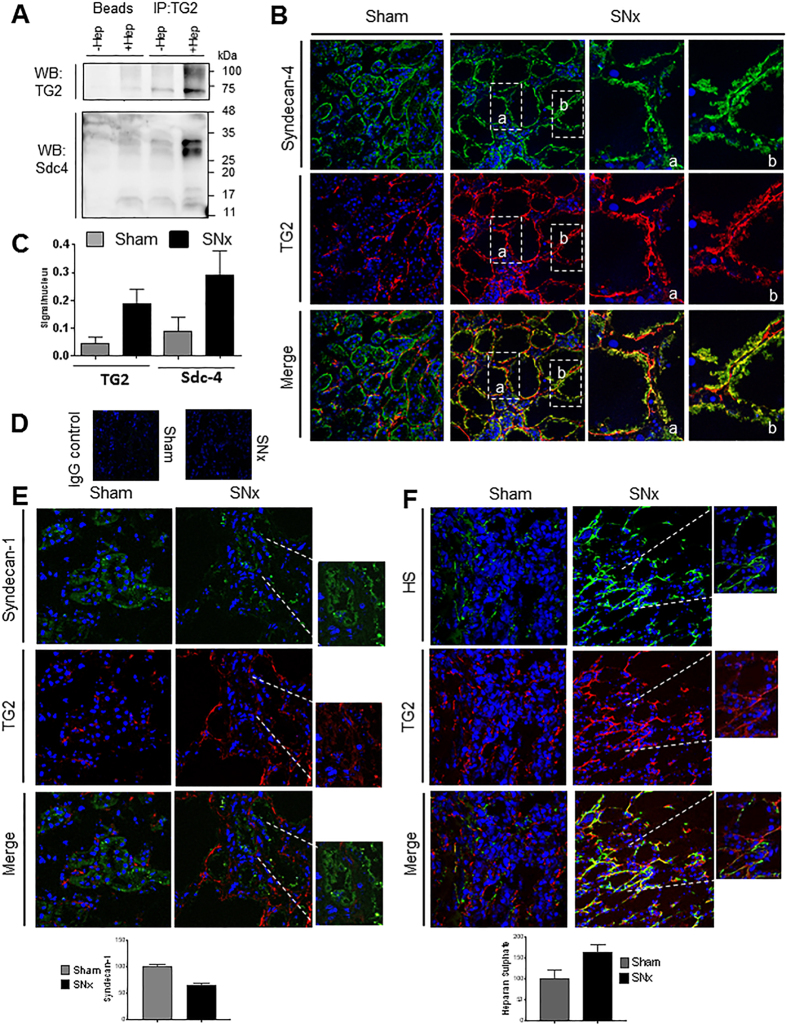
Co-association of TG2 and Sdc4 in kidneys. (**A**) Co-immunoprecipitation analysis of TG2 and Sdc4 in whole kidney lysate. TG2 was immunoprecipitated from kidney homogenate using a mouse monoclonal anti-TG2 (IA12) antibody crosslinked to protein A/G magnetic beads as reported in the Methods, after that HS chains were digested using Heparitinase I (50 mU/ml) (+Hep). Beads-only controls were also performed to confirm the specificity of the binding. Proteins were separated by reducing SDS-PAGE (12% w/v) and immunoblotted for detection of TG2 and Sdc4. (**B**–**D**) Sdc4 and TG2 immunostaining of unfixed cryostat sections. Immunostaining was performed using rabbit polyclonal anti-Sdc4 antibody and mouse monoclonal anti-TG2 antibody followed, respectively, by donkey anti rabbit Alexafluor 488, with green emission, and goat anti-mouse DyLight 594, with red emission. Representative pictures of Sdc4 and TG2 stainings are shown separately and merged for control (Sham) and SNx kidneys (advanced fibrosis) (**B**). Basolateral membrane localisation of Sdc4 and TG2 post-SNx is shown in detail using 630X magnification pictures (a,b). Sdc4 and extracellular TG2 were quantified by ImageJ intensity analysis in four kidneys per treatment (3 sections per kidney; 4–7 non overlapping images per section) and values expressed relative to the number of nuclei (**C**). In negative controls the primary antibodies were substituted by non-immune mouse and rabbit IgGs (**D**). (**E**) Sdc1 and TG2 immunostaining of unfixed cryostat sections as described in the Supplementary Methods. Mouse monoclonal anti-Sdc1 antibody and rabbit polyclonal anti-TG2 antibody followed, respectively, by goat anti-mouse IgG Dy Light 594 and donkey anti rabbit Alexafluor 488 were used. The emissions are displayed as green for Sdc1 and red for TG2 for consistency with the other stainings. (**F**) HS and TG2 immunostaining of unfixed cryostat sections with mouse IgM anti-HS 10E4 antibody and rabbit polyclonal anti-TG2 antibody followed by FITC-conjugated goat anti-mouse IgM and donkey rabbit IgG AlexaFlour 568. Representative pictures and corresponding image analysis of multiple random fields are shown for control (Sham) and SNx kidneys (advanced fibrosis). 63X magnification (detail 200X).

To investigate whether TG2 interacted with Sdc4, TG2 immunoprecipitates were probed for the presence of Sdc4 core protein, before (−Hep) and after (+Hep) cleavage of the HS chains by heparitinase I digestion. Elimination of the glycosoaminoglycan (GAG) chains revealed a band corresponding to the Sdc4 core protein homodimer^43^ in the TG2 precipitate from rat kidney lysate and eased the detection of TG2 itself (Fig. 7A). This suggests that HS chains of Sdc4 directly interact with TG2 *in vivo.* Immunofluorescence analysis of unfixed cryostat sections revealed that in normal tissues (sham-operated) TG2 had a weak basolateral membrane localisation (Fig. 7B). Post-SNx, TG2 significantly accumulated in the ECM of SNx kidneys compared to sham operated kidneys (Fig. 7B) and was found to be largely co-localised with Sdc4 in the extracellular environment (Fig. 7B, yellow-orange staining). There was only a weak intensification of the Sdc4 staining post-SNx in the tubular basement membrane and in the thickened tubulointerstitium (Fig. 7B). However, Sdc4 was higher post-SNx compared to control (sham-operated kidneys) when considered relative to the residual number of cells lining the tubules (Fig. 7C). The tubules assumed the aspect of largely cell-deprived cylinders of basement membrane, where TG2 and Sdc4 were located. Negative controls were performed in sham and SNx samples using non-immune mouse and rabbit IgGs instead of primary antibodies, displaying no background (Fig. 7D). These data are consistent with increases of TG2 (Fig. 1) and Sdc4 (Fig. 3) expression measured by qPCR in the SNx kidneys, and suggest a role for Sdc4 in recruiting and accumulating TG2 at the basolateral membrane in the SNx model of progressive kidney fibrosis. To investigate whether other syndecans and HSPG colocalise with extracellular TG2 in fibrosis we carried out a dual staining of Sdc1 and TG2, as Sdc1 was previously reported to be involved in Tubular Epithelial Cells (TEC) repair^44^. As shown in Fig. 7E, Sdc1 had a diffuse subtle staining in TEC and was concentrated in dots along the basolateral membrane but less frequently than Sdc4. The staining of Sdc1 did not increase post-SNx and Sdc1 did not co-localise with TG2, despite TG2 was abundantly secreted post-SNx. These data are in keeping with mRNA quantifications showing less Sdc1 than Sdc4, and no induction of Sdc1 post-SNx (Fig. 3). Visualisation of HS by monoclonal antibody 10E4 (Fig. 7F), which detects HS typical of the peri-cellular matrix, in particular the basement membranes^45^, showed a clear accumulation of HS post-SNx which was located in the tubular basement membrane and interstitium; TG2 largely co-stained with 10E4 HS (Fig. 7F). This suggests that TG2 can potentially bind other extracellular matrix HSPG post-SNx, although Sdc4, given its transmembrane position, more likely influences TG2 cell surface trafficking. To test whether TG2 preferentially associates with HS proteoglycans, we examined whether TG2 co-localised with versican, a chondroitin sulphate proteoglycan which in renal tissue was found expressed in areas with tubulointerstitial fibrosis in CKD^46^. Versican-immunostaining (Suppl. Fig. 2) was weak in sections from sham-operated control kidneys, however it intensified post-SNx. The antibody did not seem to stain the basolateral membrane but mainly the interstitium, and the staining was weaker compared to that of 10E4-reactive HS or Scd4 (Fig. 7B,F). Despite TG2 being abundantly externalised, no co-localisation with versican was noted (Suppl. Fig. 2).

### Latent TGF-β binding protein-1 (LTBP-1) is a substrate of cellular TG2

**Figure 8:**
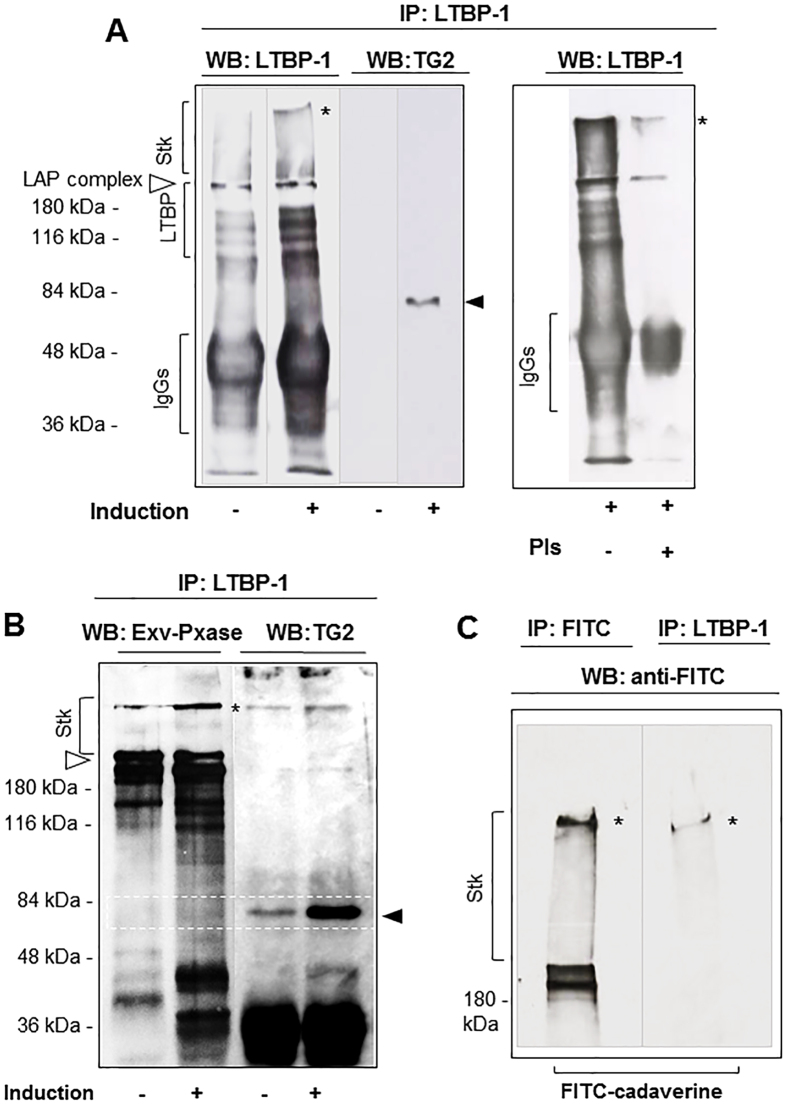
LTBP-1 co-associates with TG2 and it is incorporated in high molecular weight extracellular complexes in cells induced to overexpress TG2. Swiss 3T3 cells previously induced (+) or non-induced (−) for TG2 expression were reseeded and cultured for a further 48 hours to obtain confluent monolayers. (**A**) Cells were scraped in lysis buffer and LTBP-1 immunoprecipitated with Ab-39 antibody, as described in the Methods and Supplementary Information. Immunoprecipitates were separated by reducing SDS-PAGE (7% w/v) and subjected to Western blot for LTBP-1 and TG2. (**B**) Cells were cell surface biotinylated prior to cell lysis and LTBP-1 precipitation as described in A. Blots were revealed with extravidine-peroxidase or anti-TG2 antibody. (**C**) Cells were incubated with FITC-cadaverine in the culture medium for 15 h prior to cell lysis followed by either FITC or LTBP-1- precipitation, using anti-FITC antibody or Ab-39. Immunocomplexes were separated by reducing SDS-PAGE and subjected to Western blot for FITC to track TG substrates. Clear triangle denotes LTBP-1- LAP complex; black triangle denotes TG2; asterisk, high molecular weight complex of LTBP-1.

One proposed way through which TG2 contributes to fibrosis development is via the activation of latent TGF-β. This appears to depend on LTBP-1 cross-linking to the matrix as demonstrated employing general TG inhibitors^15^,^47^. To investigate if LTBP-1 is a substrate for cellular TG2, firstly we immunoprecipitated LTBP-1 from a cell line of Swiss 3T3 fibroblasts in which we could conveniently modulate expression of TG2 through an inducible, tetracycline (Tet)-regulated promoter^48^. In this cell line it was previously shown that induction of TG2 expression leads to an increase in biologically active TGF-β^49^. Immunoblotting (Fig. 8A) revealed typical LTBP-1 forms (in the 120–140 kDa range) and higher bands (~180–250 kDa) representing the latency associated peptide (LAP) complex form of LTBP-1 (Fig. 8A, clear triangle). These became more intense in cells induced to over-express TG2 by withdrawal of Tet (induction+) than in cells with a background level of TG2 (induction−), and were accompanied by a higher molecular weight LTBP-1-immunoreactive band at the top of the 2.5% stacking gel (Fig. 8A, asterisk). This form may represent LTBP-1 covalently associated to matrix proteins or multimerised. TG2 was found in LTBP-1 precipitates of induced cells, by immunoblotting with anti-TG2 antibody, suggesting their association (Fig. 8A, solid triangle). Plasmin digestion led to typical LTBP-1 release^50^,^51^, although the higher LTBP-1 bands were less susceptible to plasmin cleavage (Fig. 8A, +Pls). To visualise extracellular LTBP-1, cells were cell-surface biotinylated prior to LTBP-1 immunoprecipitation (Fig. 8B). Western blotting analysis with extravidin-peroxidase showed a similar profile of the higher LTBP-1 immunoreactive bands (180–250 kDa) in both non-induced cells (−) and cells induced to overexpress TG2 (+) (Fig. 8B). As noticed in Fig. 8A, cells overexpressing TG2 revealed an increased higher molecular weight LTBP-1 form (Fig. 8B asterisk), which was also weakly immunoreactive to anti-TG2 antibody, suggesting that TG2 induces matrix processing of extracellular LTBP-1. To track TG activity in LTBP-1, cells overexpressing TG2 were grown in the presence of a fluorescent amine substrate of TG transamidation (FITC cadaverine), and then immunoprecipitated with anti-LTBP-1 antibody (Fig. 8C). FITC cadaverine was found to be incorporated into the high molecular weight LTBP-1 reactive complex in the stacking gel (Fig. 8C, asterisk). These data suggest that the LTBP-1 reactive band detected in TG2-overexpressing cells (Fig. 8A–C, asterisk) results from TG2 crosslinking of LTBP-1 in the matrix.

### Extracellular TG2 induces activation of TGF-β1 in rat tubular epithelial cells in a HS-dependent way

**Figure 9:**
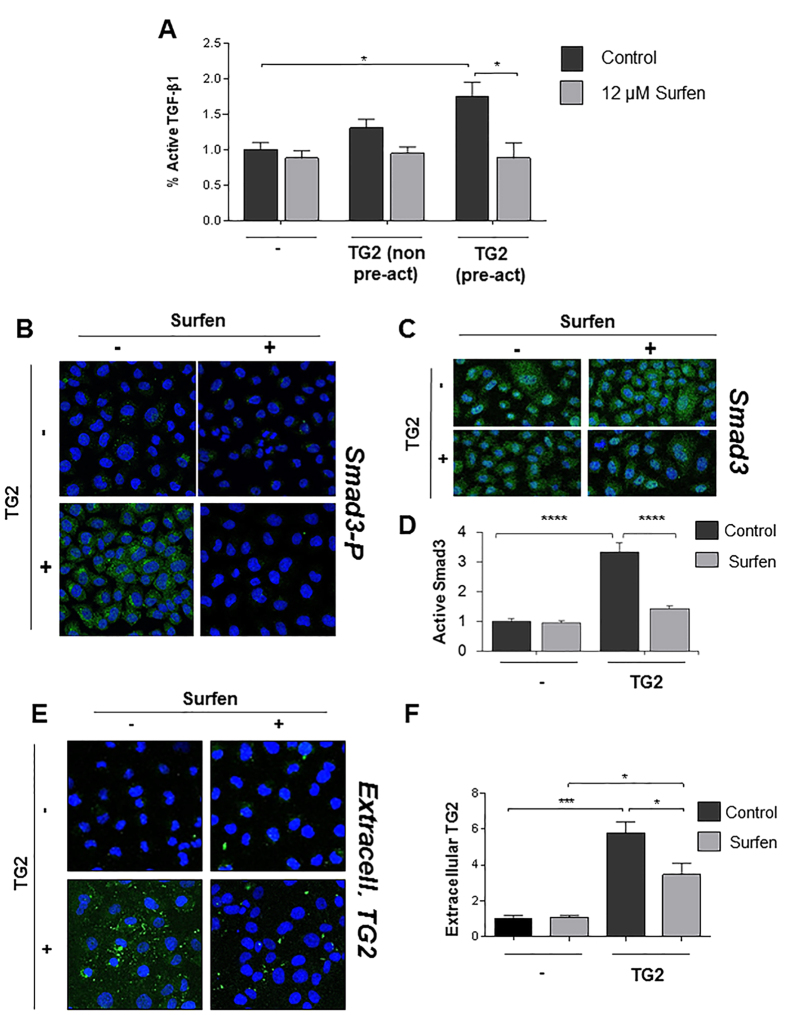
HS antagonist surfen affects TGF-β1 activation through lowering extracellular TG2. (**A**) NRK52 cells were grown in a 24 well plate and treated with HS antagonist surfen (12 μM) for 15 minutes before addition of recombinant TG2 at a final concentration of 20 μg/ml for 15 hours. Active TGF-β1 was quantified in 100 μl of conditioned medium by the MLEC -TGF-β1 reporter assay, as detailed in Supplementary Information. Data represent the mean % of active TGF-β1 expressed relative to the control without added TG2 (equalised to 1) ± SEM. A typical experiment undertaken in triplicate is shown. TG2 (non pre-act), recombinant TG2 not pre-activated by reduction. (**B**) To assess Smad3 activation, NRK52 cells were grown in an 8-well chamber slide and in some cases treated with 12 μM surfen before addition of pre-activated G2 as described above. After fixation and permeabilisation, active Smad3 was detected by rabbit anti-Smad3(pSer425) polyclonal antibody followed by donkey anti rabbit Alexa 488. (**C**) Total Smad3 was detected in replica wells by rabbit anti-Smad3 polyclonal antibody followed by donkey anti rabbit Alexa 488. Nuclei were stained with DAPI. Representative figures at 100X magnification are shown. Total Smad3 was unvaried among treatments. (**D**) Active Smad3 was quantified by ImageJ intensity analysis (8 non overlapping images per section) and presented as mean relative intensity of green over blue (DAPI) ± SEM, normalised for the total Smad3 in the corresponding treatment and expressed relative to the control without added TG2 (equalised to 1). (**E**) NRK52 cells were grown in an 8-well chamber slide and treated with 12 μM surfen as described above before addition of active recombinant TG2. After fixation, matrix bound TG2 was immunostained by a mouse monoclonal anti-TG2 antibody followed by sheep anti mouse-FITC. Nuclei were stained with DAPI. In negative controls the primary antibodies were omitted. (**F**) Cell surface and matrix bound exogenous TG2 was quantified by ImageJ intensity analysis (8 non overlapping images per section), and presented as mean relative intensity of green over blue (DAPI) ± SEM, and expressed relative to the control without added TG2 (equalised to 1).

Having shown that LTBP-1 is a substrate of TG2, we asked whether the extracellular TG2 fraction could sustain matrix activation of TGF-β1 and whether HS were implicated in this process. We developed an *in vitro* model simulating matrix TG2 accumulation in the extracellular milieu of TEC, whereby exogenous active TG2 was added to the conditioned medium of a monolayer of NRK52 rat TEC. When active TGF-β1 was measured by the mink lung epithelial cells (MLEC)-reporter assay^52^ (Fig. 9A), the conditioned medium of cells incubated with exogenous active TG2 (Fig. 9A, +TG2) displayed a significantly higher level of TGF-β1 activity than untreated cells. Non pre-activated TG2 raised the level of active TGF-β1 but not always at a significant level (Fig. 9A). As shown before, pre-activation of purified TG2 by reduction is required, as Ca^2+^ -bound TG2 is predominantly maintained in an inactive state by a disulfide bond between proximal Cys residues^25^,^53^. Therefore these findings give direct evidence for the first time that extracellular TG2 induces TGF-β1 activation. In order to investigate the role of HS-TG2 interaction in this process, we employed a small-molecule antagonist of HS chains, surfen (bis-2-methyl-4-amino-quinolyl-6-car-bamide)^54^. Pre-incubation of the NRK52 monolayer with surfen did not affect the basal level of active TGF-β1, but clearly reduced matrix TGF-β1 activation induced by extracellular TG2 (Fig. 9A). To confirm that active TGF-β1 once released from the large latent complex induces receptor complexes with kinase activation, we detected the level of phospho-Smad3 relative to total Smad3 in the same cell system (Fig. 9B–D). Extracellular TG2 induced Smad3 phosphorylation in the NRK52 TEC which was significantly reduced by pre-incubation with the HS antagonist surfen (Fig. 9B–D).

Immunofluorescence staining revealed that TG2 added extracellularly accumulated in the interstitial matrix and pericellular matrix of NRK52 cells (Fig. 9E,F) and it was catalytically active as shown by *in situ* TG2 activity (Suppl. Fig. 3A,B). Pre-incubation of the cell monolayer with surfen (Fig. 9E,F) or heparitinase (Suppl. Fig. 3C,D)) led to loss of TG2 matrix-association, and as a consequence lowered *in situ* TG2 activity (Suppl. Fig. 3A,B). These findings suggest that matrix activation of TGF-β1 by extracellular TG2 is reliant on TG2 recruitment by HS chains.

## Discussion

The ECM cross-linking enzyme TG2 has a well-established role in altering the extracellular homeostatic balance that leads to the excessive build-up of ECM in the kidney in CKD^8^,^10^,^55^. The resulting scarring is the primary pathological process associated with progressive CKD leading to end-stage kidney failure. Although TG2 belongs to a large family encoding a total of nine closely related but distinct genes^28^, little is known about the expression and biological function of the other TG isozymes in kidney. The TG family-catalytic core involved in Ca^2+^ dependent transamidation, which is responsible for matrix stabilization/deposition, is conserved in all the members, with the exception of band 4.2 protein, an inactive form with a predominant role in erythrocytes^3^. To gain insights into the role of all the TG genes in kidney, we analyzed the expression of all the TG active members in the SNx ablation model of renal scarring^10^,^35^. TG4, which has a well described prostate-restricted expression pattern^3^, was omitted from the analysis. As the disease model progressed, all the TG family members rose steadily post-SNx, peaking at a time point of “advanced fibrosis”, which corresponds to severe renal scarring and follows the increase in ECM protein transcription^35^, but TG2 was the highest expressed TG isozyme in both normal and diseased kidneys. At the final stage, corresponding to end point renal failure^35^, there was still an increased production of TG proteins; however, as the disease reached the end stage, the replacement of normal with scar tissue, and loss of epithelial and mesangial cells in favour of fibroblasts, was most likely responsible for a lower capacity of TG expression across the main isoforms. TG3, known as epidermal TG, was the second most expressed TG in kidney. TG3 is mostly found in epidermis, in the upper granular layer^56^, where it cross-links loricrin, a major cornified cell envelope protein, during the differentiation of keratinocytes into corneocytes^57^. There are no ECM substrates reported for TG3^13^ and the high expression of TG3 in kidney is a novel finding which may be due to TG3 being one of the closest isoforms to TG2 (degree of conservation 41%). TG1 had the highest fold increase of the TG family members, although its overall gene expression in CKD was less than 2% of TG2. TG1-primary role is in the terminal differentiation of keratinocytes, forming the cornified cell envelope via crosslinking the keratinocytes cytoskeleton^3^. Interestingly, increased expression of keratins was recently reported in both murine and human kidneys in connection with tubular epithelial cell stress^58^, suggesting that they might be substrates for TG1. As we have revealed its increased expression in fibrotic kidney, avoiding TG1 inhibition when attempting to block TG2 is important, as mutations in TG1 are associated with lamellar ichthyosis^3^. Furthermore, work on human skin composites with pan TG inhibitors has demonstrated severe parakeratosis and loss of the keratin layer as a consequence of TG1 inhibition^59^,^60^.

One feature of this study is the attempt to quantify the *Tgm2* variants, resulting from alternative gene splicing. Using *Tgm2* isoform-specific oligonucleotides towards rat *Tgm2_v4* (also known as tTGv1 in human cells)^33^ and towards the rat analogue of human *Tgm2_v2*, the second most prominent *Tgm2* variant (also known as TGH, Tgase S or TG2-S in human cells)^29^,^31^,^32^, our data reveals for the first time the presence of *Tgm2* variants in rat kidney and their increase in expression post-SNx. The lack of the C-terminal regulatory GTP binding site in *Tgm2_v2*, which is replaced by a new epitope formed by intron retention, makes it potentially more active and sensitive to Ca^2+^ 30. Although the TG2 variants account for a small fraction of the total level of TG2 transcripts in the rat SNx model, their over-expression during fibrosis progression may escape normal regulatory pathways and might be difficult to control.

In recent years, the HSPG-family member syndecans have emerged as binding partners for TG2 and modulators of its extracellular function^23^,^24^,^26^,^39^,^40^. Here we have shown a significant correlation between Sdc4 and TG2 expression in the SNx model of CKD. Cell surface HSPG have been found to be essential for the proliferation of renal fibroblasts, through facilitating FGF2/receptor interaction^61^, and Sdc4 was up-regulated in both IgA nephropathy^62^ and diabetic nephropathy^63^. Our work has revealed that Sdc4 is the only family member significantly induced during the progression of kidney fibrosis, despite a reported role for Sdc1 in tubular wound healing and survival^44^. The drop in Sdc1 and Sdc2 transcripts observed in the first phase of the fibrotic response is consistent with early observations that a decrease in Sdc1 causes epithelial to mesenchymal transition^64^, an event linked to fibrosis development; it is also in agreement with a decline in Sdc1 transcript caused by FGF2 recently reported in a cell line of proximal renal TEC^65^. Sdc3, being restricted to the main subdivisions of the central nervous system, was omitted from the investigation^66^. There was a clear parallel co-association between TG2 and Sdc4, as well as an increased directly adjacent staining post-SNx. The 2 proteins co-precipitated from kidney lysates and TG2 was more clearly detectable after cleavage of the immunocomplexes with heparitinase, suggesting that the HS chains mediate Sdc4-TG2 interaction. In accordance with gene expression analysis, Sdc1 was lower in expression compared to Sdc4, and it did not increase post-SNx, neither it colocalised with extracellular TG2. This is consistent with an emerging role for Scd1 in “functional repair” rather than “non-functional scarring”^44^.

Cleavage of the extracellular GAG chains of HS proteoglycans led to a complete wash out of extracellular TG2 in the rat kidney sections, consistent with previous observations that HS/Sdc4 deletion results into decreased TG2 in the tubulointerstitium^27^. Extracellular TG2 co-stained with 10E4-reacting HS in the pericellular matrix and basal membrane. Therefore, we cannot exclude TG2 interaction with HSPG other than Sdc4, although Sdc4, given its transmembrane position, more likely influences TG2 cell surface trafficking. There was no co-localisation between TG2 and the chondroitin sulphate proteoglycan versican despite this being expressed in kidney post-SNx^46^, showing that TG2 preferentially interacts with HSPG.

We then asked the new question whether HS affinity was a unique feature of TG2 among the TG family members. Solid binding assays in real time revealed a surprisingly high interaction between TG1 and heparin, a synthetic analogue of HS. However, sequence analysis ruled out the presence of the conformation-dependent high affinity heparin binding site previously reported for TG2, formed by amino acid cluster RRWK_262–265_ combined with cluster KQKRK_598–602_^22^. None of the other proposed heparin binding sites of TG2 had a homology in TG1^39^,^40^, but a high level of basic amino acids, including a potential heparin binding site (RSRR_33–36_), was noted in the N-terminal 10 kDa domain of TG1, known to be modified by myristoylation for membrane anchorage, but cleaved during keratinocyte differentiation^38^. However, it is worth noting that TG1 forms large oligomers, as shown by structural studies (PDB entry 2XZZ), likely explaining the high binding response observed in SPR. Other TG family isoenzymes displayed a lower level of heparin binding relative to TG2, with TG3 being the most similar to TG2 although with a weaker affinity. When considering the TG2 variants, Tgm2_v2 is anticipated to lack the crucial basic amino acid cluster at position 598–602, hence it is predicted not to form the conformational binding site reported for the main TG2^22^. Moreover, both Tgm2_v2 and Tgm2_v4 lack K_633_ (which corresponds to K_634_ in human TG2), another important residue for the affinity of TG2 to heparin^22^. Therefore our study suggests that TG2, the main TG in fibrotic kidney, builds up in the matrix in association with the HSPG through a unique interaction, which is missing in the other family members and in the truncated variants of TG2.

Next we looked at the consequences of TG2 externalization and its interaction with HS in fibrosis development. We showed that extracellular TG2 induces the activity of TGF-β1 in a tubular epithelial cell line largely employed in renal cell studies^67^. TGF-β1 is a central mediator of progressive renal fibrosis by inducing fibroblasts and myofibroblasts proliferation, epithelial to mesenchymal transition and matrix synthesis^1^,^18^. The action of TGF-β1 is tightly regulated in a special way to prevent TGF-β1 signaling dysregulation, as overexpression of TGF-β1 alone causes multiple organ fibrosis^17^. It exists in the ECM of most cells in a large latent form, consisting of TGF-β1 homodimer non covalently attached to its latency propeptide (LAP), which in turns is covalently bound via disulfide bonds to LTBP, a fibrillin-like ECM protein family, of which LTBP-1 is the best characterized. Consistent with the idea that recruitment of large latent TGF-β1 and immobilization in the ECM via LTBP is a prerequisite to cell-mediated TGF-β1 activation^17^,^68^, here we have shown that inducible overexpression of TG2 in a cell line of Swiss 3T3 fibroblasts leads to matrix recruitment of large latent TGF-β1 and that LTBP-1 is a substrate for extracellular TG2 in cells in culture. Although there is convincing evidence in the literature including our own work to suggest that TG2 activates TGF-β1 *in vitro* and *in vivo*^16^,^49^,^69^, to our knowledge this is the first time that extracellular TG2 has been shown to be sufficient to mediate this process. TG2 is secreted by tubular epithelial cells^9^,^10^,^67^, but matrix accumulation of extracellular TG2 is also contributed by other cells types including fibroblasts, migrating into the site of injury, and endothelial cells which are a rich source of TG2^3^. Deposition in the ECM, leads to increased stability of TG2 and protection from proteolytic degradation^70^. Here we hypothesized that HS regulates TGF-β1 activity by affecting TG2 matrix deposition. We showed that antagonism of HS greatly impairs the increase in TGF-β1 activity and TGF-β1 receptor stimulation (phosphorylation of Smad3) induced by extracellular TG2 in the tubular epithelial cell line, without affecting the basal level of TGF-β1. Previously we showed that Sdc4 KO resulted in lower TGF-β1 activation and TG2 accumulation and this was accompanied by amelioration of fibrosis scores in the AAN mouse model of CKD^27^. Others have shown that HS mediate LTBP binding to fibronectin via LTBP affinity for heparin^71^, therefore HS would impact on TGF-β1 activation in this way. We have now clarified the interplay between HS and extracellular TG2 in TGF-β1 activity. Our new data implies that HSPG promote fibrosis development also indirectly, via a mechanism involving control of TG2 deposition in the matrix, which in turn impinges on matrix activation of TGF-β1. Therefore it is possible that HS promote matrix targeting of large latent TGF-β1 by both recruiting TG2 and LTBP-1 to the ECM.

**Figure 10:**
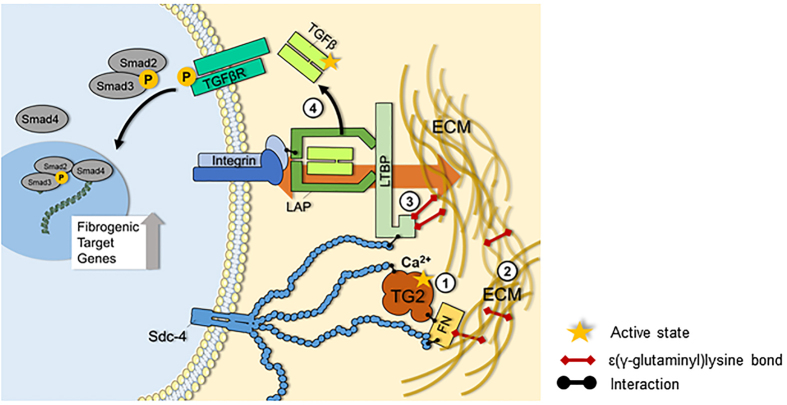
Interplay of TG2 and HS in the mechanical activation of latent TGF-β1. TG2 is released from cells in the progression of kidney fibrosis and cell surface HS are critical for its extracellular recruitment. By calcium-dependent transamidation, extracellular TG2 increases the general degree of cross-linking of the ECM (1), produces a remodelled and stiffened matrix typical of the fibrotic condition (2) and incorporates LTBP-1 in the ECM, storing large latent TGF-β1 (3). These events are a pre-requisite for the mechanical release (depicted by the background orange arrow) of soluble TGF-β1 dimer from large latent TGF-β1 complex, occurring via LAP- integrin binding on the cell side, and LTBP-1 binding to a sufficiently remodelled ECM on the extracellular side. Under these conditions cell contraction will result into TGF-β1 release, and consequently engagement with its receptor (4). HS have been reported to mediate LTBP-1 association to the matrix. We have given new evidence that antagonism of HS lowers the recruitment and retention of TG2 in the matrix and greatly reduces activation of TGF-β1 by extracellular TG2.

Recent thinking ascribes to the matrix a key mechanical function in both the control and activation of TGF-β1. ECM binding of the large latent TGF-β1 complex via LTBP-1 on one hand, and αv integrin binding via LAP on the other hand, together with sufficient matrix remodeling provide mechanical tract that would lead to a change in LAP resulting in active TGF-β1-release^17^. In this process TG2 and HS are anticipated to play an interactive role which favors release of active TGF-β1 dimer (Fig. 10). TG2, externalized and immobilised by HS and fibronectin at sites of damage, grabs LTBP1 and catalyzes the incorporation of LTBP-1 in the ECM. Fibronectin^69^ and fibrillin^51^ are possible cross-linking partners for LTBP-1 matrix incorporation. Furthermore TG2 contributes to matrix remodeling and also acts as a structural adhesive protein bound to fibronectin^25^,^26^. Within the large latent TGF-β1 complex, the combination of LTBP1 immobilisation by TG2 on the matrix side together with TG2-mediated matrix remodelling, and LAP binding to integrin on the cell side, likely contribute to create the mechanical conditions for alterations in integrin-bound LAP leading to release of active TGF-β1 (Fig. 10).

HS recruit/distribute TG2 in the matrix, since our data suggest that antagonism of HS has a significant effect on extracellular TG2-mediated TGF-β1 activation. Reducing the degree of cross-linking of the matrix and controlling the role of proteoglycans are attractive strategies for lowering TGF-β1 activation in anti-fibrosis therapy. As we have shown that TG2 is so abundant in the fibrotic kidney and it affects TGF-β1 activity in a HS-dependent manner, one way to control fibrosis progression could be by inhibiting the association of HS/Sdc4 with the conformational heparin binding site of TG2.

## Methods

### Experimental animals and cell lines

All experimental procedures were carried out under licence in accordance with regulations laid down by Her Majesty’s Government, UK (Animals Scientific Procedures Act ASPA, 1986), and were approved by the University of Sheffield Animal Ethical Review Committee (ASPA Ethical Review Process). We utilised archival material from two 5/6^th^ subtotal nephrectomy (SNx) rat models of progressive renal scarring reported previously. The studies consisted of groups of five to six rats subjected to SNx or a sham operation harvested at increasing time points post nephrectomy^35^,^72^. Masson’s Trichrome staining of kidney sections showed comparable levels of tubulointerstitial fibrosis in a classical 90 day SNx study run in 2002^35^ with an accelerated 60 day SNx study^72^ (Suppl. Fig. 4). Rat tubular epithelial cells (NRK52) and Swiss 3T3 fibroblasts expressing TG2 (clone TG3) were cultured and maintained as described^48^,^67^. The two SNx rat models were utilised in line with the principle of the “3Rs” (Replacement, Reduction and Refinement) framework for humane animal research.

### Quantitative reverse transcription PCR (qRT-PCR)

Total RNA (2 μg) was isolated from the kidney of the SNx model^35^, using Trizol^TM^ (Invitrogen) and reverse transcribed with random hexamers (Promega), using Superscript reverse transcriptase (Invitrogen). The cDNA was amplified by qPCR using iQ SYBR Green Supermix (BioRad), employing the oligonucleotide primer pairs shown in Suppl. Table 1. Absolute quantifications and normalization to housekeeping genes was performed as described in the Supplementary Information.

### Kidney homogenates and western blotting of TG family members

Snap-frozen tissue from SNx and sham operated kidneys^72^ (100–200 mg) was homogenised on ice by Ultra Turrax T25 Homogeniser (IKA) in lysis buffer (0.32 M sucrose, 50 mM Tris-HCl, 2 mM EDTA, pH 7.4) containing protease inhibitors. Protein lysate (60 μg) was resolved by 10% SDS-PAGE under reducing conditions, and immunoblotted as previously described^24^. Protein detection and quantification, including primary antibodies, are described in the Supplementary Information.

### Surface plasmon resonance based binding assay

Size defined heparin (9 kDa) or HS purified from porcine intestinal mucosa (Celsus Laboratory) was biotinylated at the reducing end and immobilized on a streptavidin-functionalized CM4 Biacore sensorchip to a level of, respectively, 50 or 45 resonance units (RU), as described^22^. TG samples were diluted in HBS-EP buffer (10 mM Hepes, 150 mM NaCl, 3 mM EDTA, 0.05% P20, pH 7.4), maintained at 25 °C, and injected over both reference and heparin surfaces at a 20 μl/min flow rate for 5 min after which the formed complexes were washed with running buffer. The sensorchip surfaces were regenerated with a 1 min pulse of 0.1% SDS and 4 min pulse of 2 M NaCl.

### Immunostaining of TG2 and Sdc4 in kidney cryosections

Cryostat sections (14 μm-thick) were blocked in PBS pH 7.4, containing 3% w/v BSA, 0.1% v/v IGEPAL and 5% v/v blocking serum (donkey and goat) and incubated with 10 μg/ml primary anti-Sdc4 antibody (Zymed) and 1:75 (v/v) mouse monoclonal anti-TG2 antibody (Cub7402, Abcam), for 15 h at 4 °C in blocking buffer. In negative controls non-immune mouse and rabbit IgGs were used. After washing, the sections were incubated for 1 hour at 37 °C with 1:200 (v/v) secondary antibodies (donkey anti rabbit-Alexa Fluor 488 and goat anti-mouse DyLight 594 (Invitrogen)), in PBS with 1% w/v BSA. Slides were washed, coverslips mounted using DAPI-containing Vectashield (Vectorlabs) and visualised by laser scanning Leica SP5 confocal microscope (40X or 63X oil immersion). WCIF ImageJ integrated density tool (pixel intensity) was used for quantifications. Cells were counted via the particle size tool of ImageJ. A co-localisation plug-in of Leica SP5 confocal microscope was used to evaluate dual color overlap. All images were processed for background noise subtraction before analysing for intensity. Enzymatic pre-treatments were performed with heparitinase I, 50 mIU/ml (Iduron), for 2 hr at 37 °C.

### Immunoprecipitations

Immunoprecipitations from whole kidney lysates were carried out by using the Pierce Crosslink Magnetic IP/Co-IP Kit from Thermo Scientific, according to manufacturer’s instructions, and from cell lysates as previously described^24^. A detailed description is provided in the Supplementary Methods.

### Assay of active TGF-β1

Active TGF-β1 was measured by the MLEC -TGF-β1 reporter assay^52^, a gift from M Griffin (Aston University), as previously described^27^. Cells were seeded at 1.5 × 10^5^ cells/well in a 24 well plate in complete medium with serum. After 24 hours, the growth medium was replaced with DMEM supplemented with 2% (v/v) FBS and cells were allowed to grow for further 24 h. In some instances cells were grown in the presence of human Rh-TG2 (20 μg/ml, Zedira) (which had been pre-treated with DTT at the final concentration of 2 μM). In some cases the growth medium was supplemented with HS chains antagonist surfen, a gift from JD Esko (University of California, San Diego), used at 12 μM, a concentration which interferes with TG2-HS binding^24^. Active and total TGF-β1 were respectively assessed in 100 μl of native or acid-treated conditioned medium, which was applied as a culture medium to the MLEC of the TGF-β1 reporter system (5 × 10^4^ cells/well in a 96-well plate). After 22 h, the cells were washed twice with PBS, pH 7.4, and lysed in 1x Reporter Lysis Buffer (Promega). Light emission was measured by Polarstar Optima (BMG Labtech, Ortenberg/Germany) luminometer.

### Statistical analysis

Comparisons were made by analysis of variance (Regular 2-way ANOVA, Bonferroni’s post-test) and values were considered to be significant at p < 0.05 (*), p < 0.01 (**), p < 0.001 (***) or p < 0.0001 (****).

## Additional Information

**How to cite this article**: Burhan, I. *et al*. Interplay between transglutaminases and heparan sulphate in progressive renal scarring. *Sci. Rep.* **6**, 31343; doi: 10.1038/srep31343 (2016).

## Supplementary Material

Supplementary Information
