# Supplementary material for: Interplay between transglutaminases and heparan sulphate in progressive renal scarring
Source: Sci Rep. 2016 Oct 3;6:31343. doi: 10.1038/srep31343 (PMC5046136; doi:10.1038/srep31343)
Supplement: Supplementary Information [file srep31343-s1.pdf]

Supplementary Information

Interplay between transglutaminases and heparan sulphate in progressive renal scarring

Izhar Burhan, Giulia Furini, Hugues Lortat-Jacob, Adeola G Atobatele, Alessandra Scarpellini, Nina Schroeder, John Atkinson, Mabrouka Maamra, Faith H Nutter, Philip Watson, Manlio Vinciguerra, Timothy S Johnson, and Elisabetta AM Verderio

Suppl. Table 1

Gene Symbol	Gene Name	Accession Number	Primer sequences (5'→3')	Size
Gapdh	**Glyceraldehyde-3 phosphate dehydrogenase**	NM_017008.3	Fw: CGTCTTCACCACCATGGAGA<br>Rv: CGGCCATCACGCCACAGCTTT	300 bp
Ppia	**Peptidylprolyl isomerase A or Cyclophilin-A**	NM_017101.1	Fw: ACGCCGCTGTCTCTTTTC<br>Rv: CTTGCCACCAGTGCCATTAT	262 bp
Tgm1	**Transglutaminase 1**	NM_031659.1	Fw: TGTTGCTCAATGTCTCAGGC<br>Rv: TGACACCGTGTACTTGGGAA	351 bp
Tgm2	**Transglutaminase 2**	NM_019386.2	Fw: GTATGATGCGTCCTTCGTGT<br>Rv: CAGTTTGTTCAGGTGGTTGG	235 bp
Tgm3	**Transglutaminase 3**	NM_001108959.1	Fw: TGGCAGTAGGCAAAGAAGTC<br>Rv: CACATCGATTTTGAGGCTGC	461 bp
Tgm5	**Transglutaminase 5**	XM_001080153.1	Fw: CTTCCCTTCGACCCAGTGAT<br>Rv: GAGTGATGCTGGCTTTGTTA	561 bp
Tgm6	**Transglutaminase 6**	XM_230601.4	Fw: AGCCGGTAGCAGAGATCTT<br>Rv: AATCCTCCCCAAGTTCCTT	526 bp
Tgm7	**Transglutaminase 7**	XM_001080162.1	Fw: CAAGCAAATTCCATCACCTCCA<br>Rv: TGAGCCGGCAGCATTTCTG	470 bp
F13a1	**Coagulation factor XIII, A1 polypepetide**	NM_021698.2	Fw: CTGTTGGATTTGGAGGATGG<br>Rv: GCTCCATACATCAGGGCAGT	349 bp
Tgm2_v2	**Transglutaminase 2 variant 2**	*Predicted from human* NM_198951.1	Fw: ATGGGTCTGTGCTCAAATCC<br>Rv: AAGAAAGAACATTTGGCCCTG	488 bp
Tgm2_v4	**Transglutaminase 2 variant 4**	*Monsonego et al, 1997*	Fw: ACTTTGACGTGTTTGCCCAC<br>Rw:GCTGAGTCTGGGTGAAGACACAG	414 bp
Sdc1	**Syndecan-1**	NM_013026.2	Fw:AGGTGCTTTGCCAGATATGACT<br>Rv: CTCTTTGATGACAGAAGTGCCT	433 bp
Sdc2	**Syndecan-2**	NM_013082.3	Fw: GACATGTACCTTGACAGCAGC<br>Rv: CTCCTTAGTGGGTGCCTTCTG	510 bp
Sdc4	**Syndecan-4**	NM_012649.2	Fw: GAGTCGATTCGAGAGACTGA<br>Rv: AAAAATGTTGCTGCCCTG	366 bp

Suppl. Fig. 1

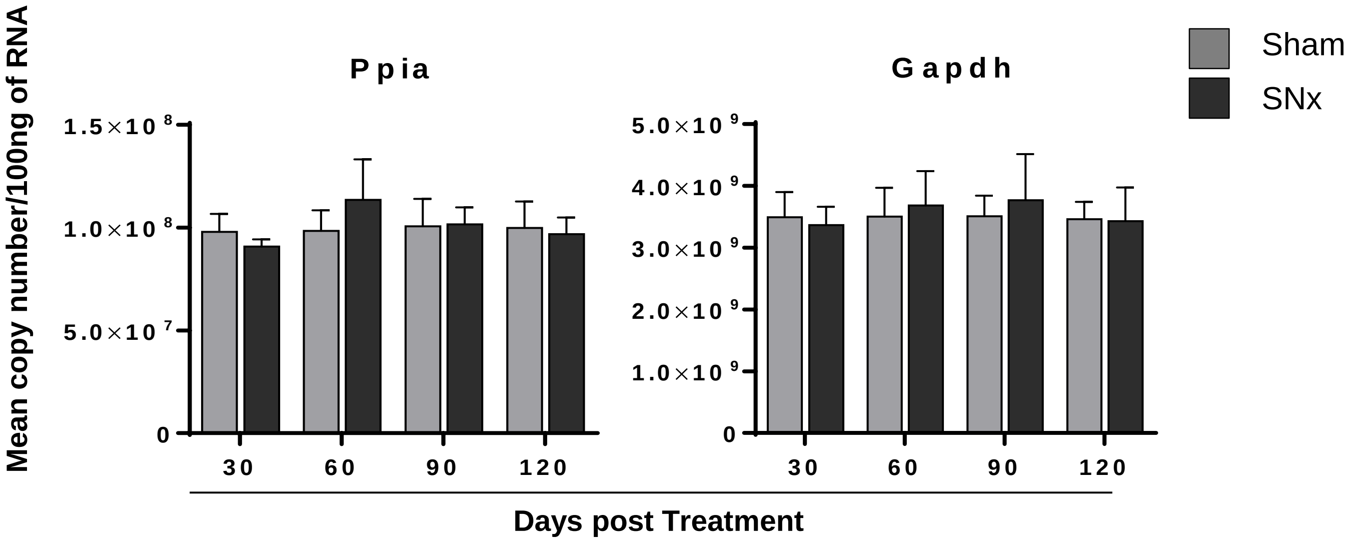

Mean copy number/100ng of RNA
Ppia
Gapdh
Sham
SNx
1.5×10⁸
1.0×10⁸
5.0×10⁷
0
5.0×10⁹
4.0×10⁹
3.0×10⁹
2.0×10⁹
1.0×10⁹
30
60
90
120
Days post Treatment

Suppl. Fig. 2

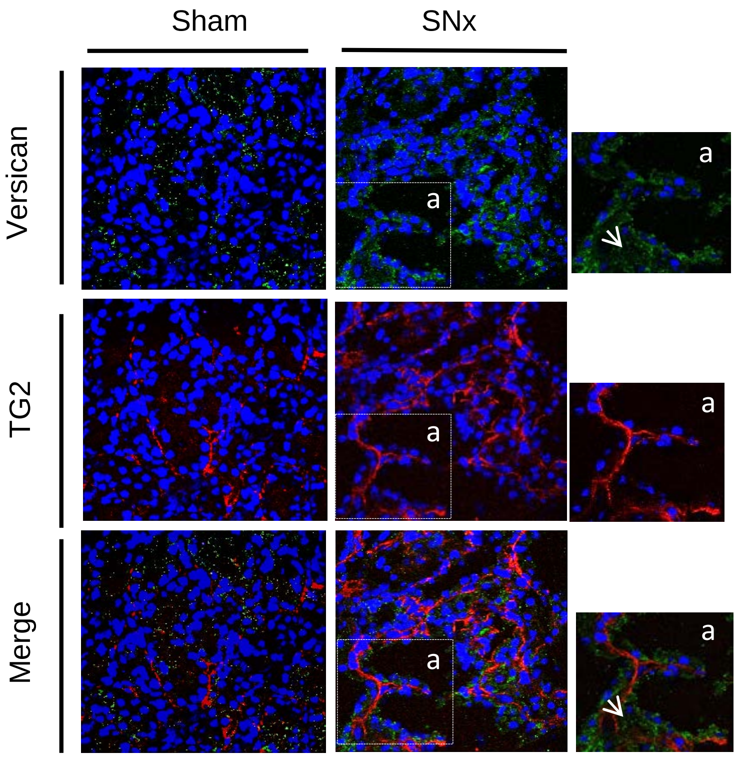

Suppl. Fig. 3

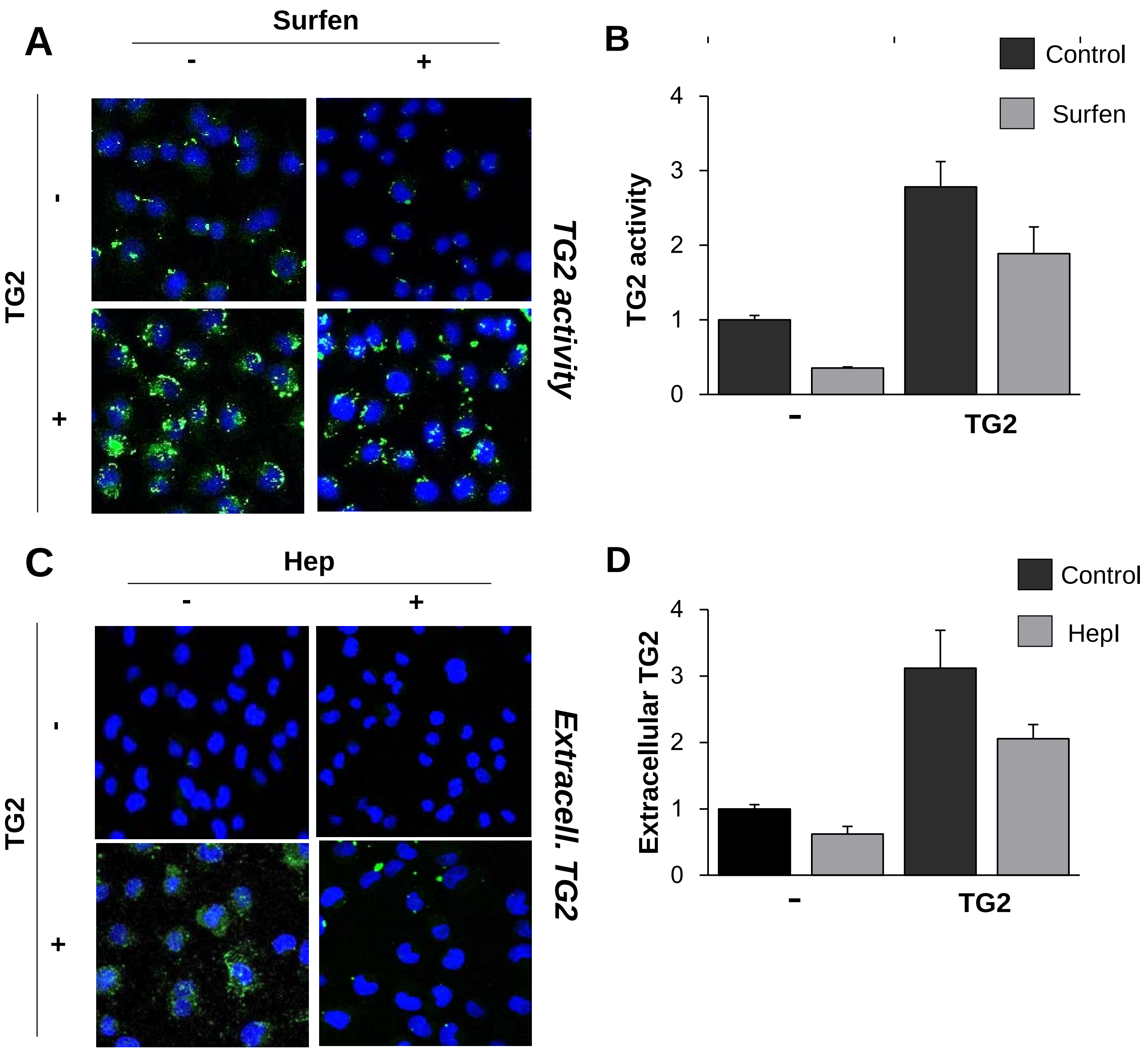
A
Surfen
-
+
TG2
-
+
TG2 activity
B
Control
Surfen
TG2 activity
4
3
2
1
0
-
TG2
C
Hep
-
+
TG2
-
+
Extracell. TG2
D
Control
HepI
Extracellular TG2
4
3
2
1
0
-
TG2

Suppl. Fig. 4

Day 90 kidneys from Exp Nephrol 2002;10:182–195

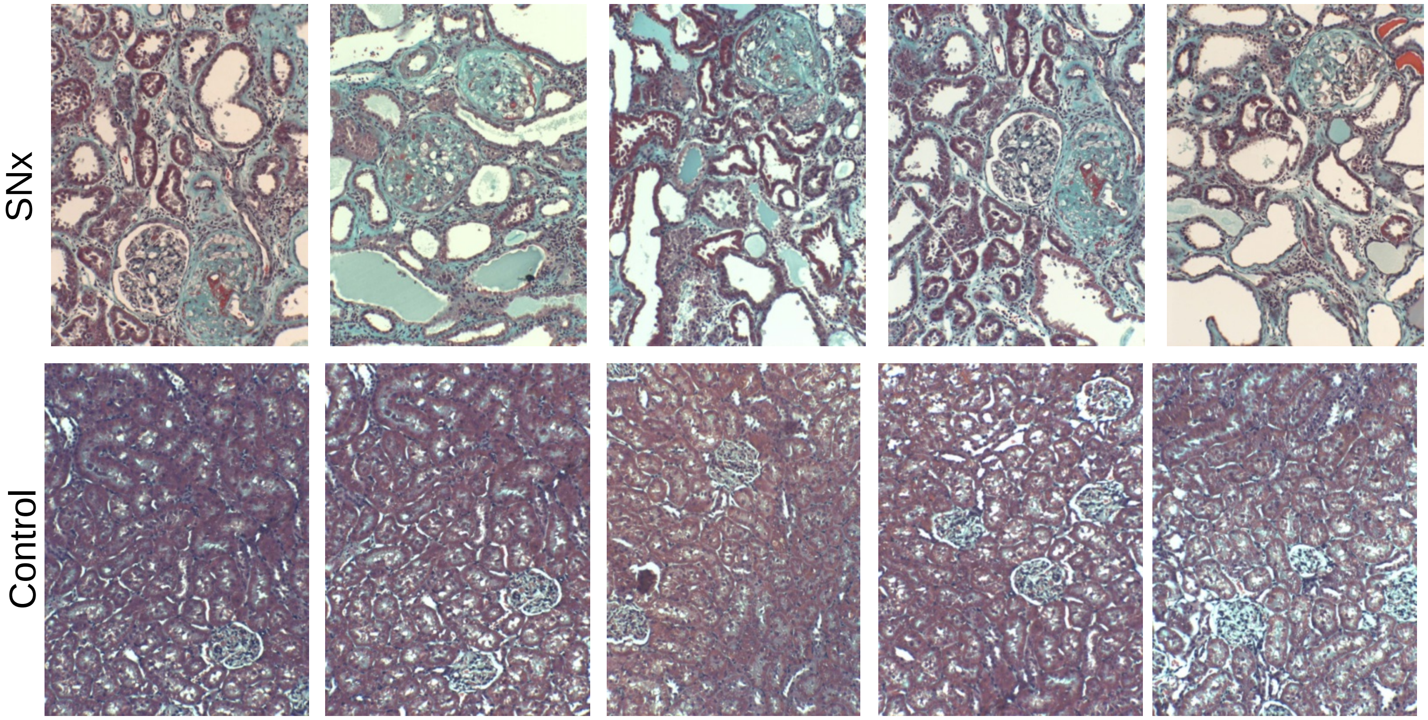

Day 60 kidneys from J Am Soc Nephrol. 2015, 26(8):1925-37

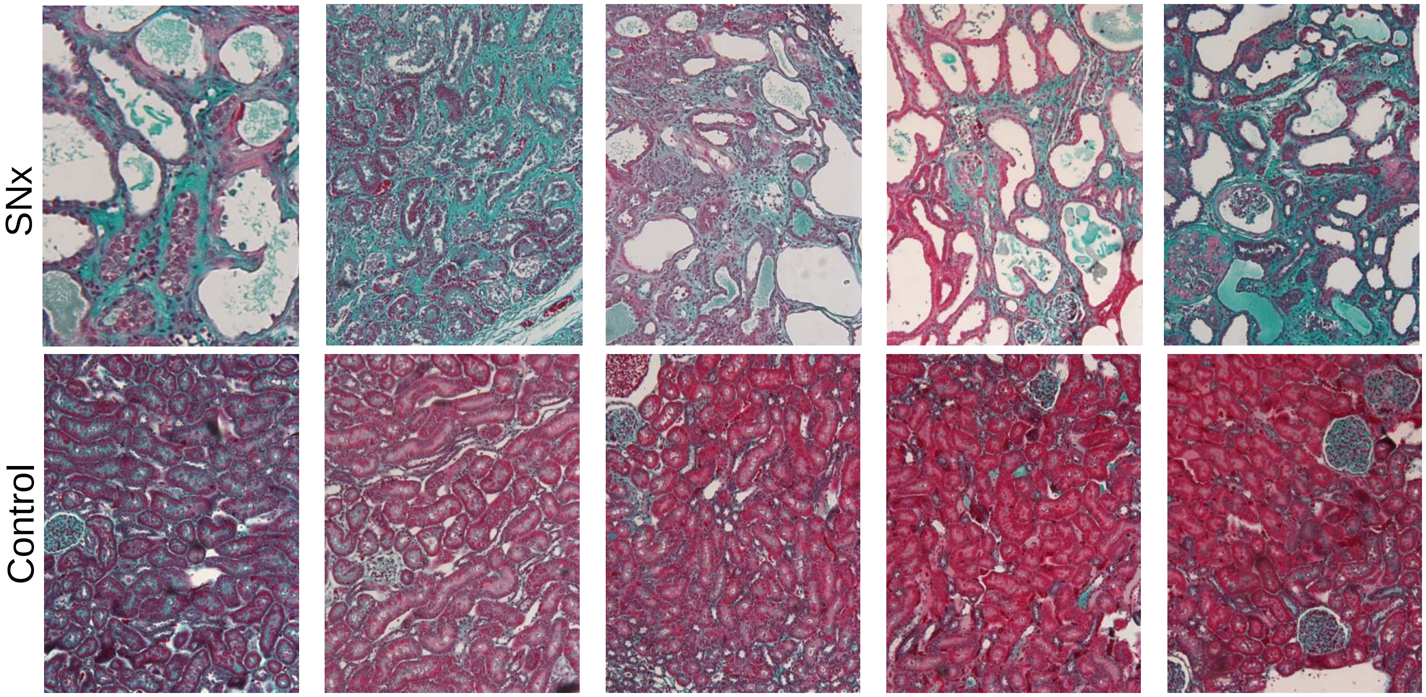

Figure legends

Suppl. Table 1. List of oligonucleotide primers used for qRT-PCR.

Suppl. Fig. 1. Expression of house-keeping genes Ppia and Gapdh at days 30, 60, 90 and 120 post-SNx and corresponding control kidneys

Data are expressed as mean copy number/100 ng RNA ± SEM. At least four kidneys were tested per time point.

Suppl. Fig. 2. Versican and TG2 dual immunostaining

Versican and TG2 immunostaining of unfixed cryostat sections with rabbit monoclonal anti-versican antibody and mouse monoclonal anti-TG2 antibody IA12 followed by donkey anti-rabbit IgG AlexaFlour 488 and foat anti-mouse IgG Dy Light 594 . Representative pictures of control (sham) and SNx kidneys (advanced fibrosis) are shown.

Suppl. Fig. 3. HS antagonist Surfen and Heparitinase reduces the level of exogenous TG2 retention in the extracellular space of NRK52 cells

NRK52 cells were incubated with RhTG2 in 8-well chamber slides as described in legend to Fig 9. (A-B) Selected wells in triplicate were treated with 12 µM Surfen for 15 minutes before addition of reduced human Rh-TG2 and FITC-conjugated cadaverine (0.5mM), to visualise in situ TG2 activity for 6 hr. Cells were fixed with 90% methanol in PBS pH7.4, and nuclei stained with DAPI. (C-D) Selected wells in triplicate were treated with with 30 mU/ml Heparitinase I (Hep I) for 1 hr before addition of reduced human Rh-TG2 for 1 hr. The level of TG2 protein was detected by immunostaining as described in legend to Fig 9.

TG2 activity (B) or protein (D) was quantified by ImageJ intensity analysis (8 non overlapping images per section) and presented as mean relative intensity of green over blue (DAPI) ±

SEM, expressed relative to the control without added TG2 (equalised to 1). Representative figures at 100X magnification are here shown.

Suppl. Fig. 4. Comparison of fibrosis between two Subtotal Nephrectomy (SNx) studies Masson's Trichrome staining of kidney sections showing comparable levels of tubulointerstitial fibrosis in a classical 90 day SNx study run in 2002 with an accelerated 60 day SNx studies performed 2015. Both studies show similar levels of tubular atrophy, interstitial expansion, cell infiltration and loss of normal renal architecture. 200 x magnification.

Detailed Methods

PCR primers, QPCR amplification and analysis

Primers (Sigma Genosys) were designed manually or using the Primer3 software (available at SDSC Biology workbench 3.2; http://workbench.sdsc.edu/), and their level of secondary structure evaluated by the DNA calculator software (available at http://www.sigma-genosys.com/calc/DNACalc.asp). All primer-set were planned to amplify genes across intervening sequences whenever possible (Suppl. Table 1). Specificity of the primers was confirmed by melting point analysis and end-point analysis of PCR products (gel electrophoresis) showing that all fragments were of the anticipated size (Suppl. Fig 1). To rule out PCR amplification of contaminating genomic DNA, not reverse transcribed RNA samples were included in each PCR reaction. QPCR reactions were performed in a Corbett Rotor-Gene^{TM} 6000 rotary analyzer (initial denaturation: 95°C, 3 min; followed by 40 cycles: 95°C, 30 sec; 58-63°C, 30-45 sec; 72°C, 20-45 sec, depending on the transcript to be amplified). For all transcripts studied, a ramp temperature from 72°C to 95°C was used to generate the melt curves, which were used to check the homogeneity of the amplified transcripts. Each sample was run in triplicate and a no-template control was included to rule out contamination. Absolute quantifications were performed using standard curves of transcript-specific RT-PCR products, purified from low melting gel (0.7% W/V, Bioline) using SpinX columns (Corning), according to the manufacturer's instructions. The gel-purified cDNA standards were quantified spectrophotometrically at 260nm using NanoDrop 8000 (Thermo Scientific) and the copy number calculated from the concentration using the Avogadro number (6.02×10^{23} mol^{-1}). Serial dilutions of the transcript-specific cDNA standards with known amounts of input copy (10^{10}-10^{2} molecules) were amplified by real time PCR in triplicate and the corresponding threshold cycle values plotted against the log copy number to generate standard curves. The reaction efficiency ($E= [10^{(-1/M)}] – 1$), slope (M) and r squared (r^2) were determined by the Corbett Rotor-Gene 6000 series software (optimal values for reaction efficiencies, slope and r

squared are 1, –3.322 and 1 respectively). In each experimental set, each target kidney sample was analysed by real time RT-PCR for *Ppia* and *Gapdh* expression. To normalize for RNA input and possible inefficiencies in cDNA synthesis copy numbers obtained for the target genes (TG and Syndecan family) were divided by the corresponding Ppia value. Absolute quantifications were performed using standard curves of purified RT-PCR products which showed high test linearity (r, 0.991-0.999) and efficiency (0.63-1.1) over the wide quantification range (10^2 -10^8 molecules).

Western blotting of TG family members

The following primary antibodies were used to detect the TG members at the stated dilutions in blocking buffer (5% non-fat milk in TBST): goat polyclonal anti transglutaminase-1 (N20, Santa Cruz Biotechnology) 1:500 (v/v); goat polyclonal anti transglutaminase-3 (C-19, Santa Cruz Biotechnology) 1:500 (v/v); goat polyclonal anti transglutaminase-6 (G-16, Santa Cruz Biotechnology) 1:500 (v/v); goat polyclonal anti transglutaminase-7 (L-14, Santa Cruz Biotechnology) 1:500 (v/v); mouse monoclonal anti transglutaminase-2 (IA12, obtained from Sheffield University) 1:2000 (v/v). Cyclophilin-A and β-Tubulin were revealed as loading control using respectively mouse monoclonal anti β-Tubulin (Sigma) 1:2000 (v/v) and rabbit polyclonal anti cyclophilin A (ab41684, Abcam) 1:1000 (v/v). Proteins were revealed by enhanced chemiluminescence, after incubation with appropriate HRP-conjugated secondary antibody (Goat anti-mouse IgG, DAKO, 1:1000 (v/v); Goat anti-Rabbit IgG (Sigma) 1:2500 (v/v)). Quantitative comparison of protein band intensity was obtained by Aida Image Analyser v.4.03 (Raytest), according to the manufacturer's guidelines.

Immunoprecipitations

Immunoprecipitations from whole kidney lysates were carried out by using the Pierce Crosslink Magnetic IP/Co-IP Kit from Thermo Scientific. Mouse monoclonal anti TG2 antibody (IA12) [27] was allowed to bind Protein A/G magnetic beads for 2 hr in constant rotation at room temperature, then cross-linked to the beads with 20 uM disuccinimidyl suberate (DSS) in

dimethyl sulfoxide (DMSO), for 4 hr in constant rotation. Equal amounts of total proteins (1.5 mg tissue lysate) were applied to the beads for 15 h at 4°C in constant rotation to allow the antigen to bind, then the bound antigen was eluted according to the manufacturer's instructions. Beads with no conjugated antibody were a negative control. In selected elutions, HS chains of the HSPG were digested using Heparitinase I (50 mIU/ml) for 2 hr 37°C under constant rotation. Equal volumes of TG2-immunoprecipitates were separated by reducing SDS-PAGE (12%w/v) and immunoblotted for detection of TG2 using 1:1000 (v/v) Anti-TG2 antibody (Ab80563 Abcam) and Sdc4 using 1:500 (v/v) anti-Sdc4 antibody (Ab24511 Abcam). Immunoprecipitations from cell lysates was performed as previously described [14]. Specifically, confluent cell monolayers (grown for 48h) were washed in ice cold PBS, pH 7.4 containing 3 mM EDTA and solubilised in lysis buffer (10 mM Tris HCl (pH7.4) 150 mM NaCl, 0.5% NP-40 and 1% TritonX100, 1 mM EDTA, 1mM EGTA, 0.2 mM sodium vanadate) supplemented by protease inhibitors by cell scraping. After three rounds of brief sonication on ice, the total cell lysate was pre-cleared with protein-G-agarose incubation for 3 hr at 4°C. After low speed centrifugation (to remove remaining cells and nuclei), lysates were incubated with rabbit anti-LTBP-1 antibody (Ab-39, BD Biosciences Pharmingen). The immunocomplexes were separated by reducing SDS-PAGE (7% polyacrylamide) and immunoprobed with anti-TG2 antibody or as a control anti-LTBP-1 antibody. In some instance cells were incubated with FITC-cadaverine (0.4 mM) for 15h in the culture medium prior to cell lysis and LTBP precipitation, to enable tracking of TG cross-linking, and labelled substrates identified by anti-FITC antibody. In some cases cells were cells surface biotinylated (Boehringer) following manufacturer's recommendations, prior to cell lysis and LTBP precipitation, and immunoprecipitates identified by streptavidin peroxidase. Bands were revealed by enhanced chemiluminescence after incubation with appropriate HRP-conjugated secondary antibody.

Analysis of active TGF-β1 in NRK52 cells with and without HS antagonism

Active and total TGF-β were determined using the MLEC luciferase TGF-β quantitative bioassay, provided by Prof Martin Griffin (Aston University) [48]. Cells were seeded at 1.5 × 10^5

cells/well in a 24 well plate in complete medium with serum. After 24 hours, the growth medium was replaced with DMEM supplemented with 2% (v/v) FBS and cells were allowed to grow for further 24 h. In some instances cells were grown in the presence of Rh-TG2 (20 µg/ml, Zedira) which was kept in a reduced active state by supplementation of DTT at the final concentration of 2 mM. In some cases the growth medium was supplemented with HS chains antagonist surfen, kindly provided by JD Esko (University of California, San Diego), used at 12 µM, a concentration which interferes with TG2-HS binding [14]. Supernatants were collected after 24 hours and acid treated to activate latent TGF-β1. Active and total TGF-β1 were assessed using 100 µl of supernatant or acid-treated supernatant applied to the MLEC ($5x10^4$ in a 96-well plate) as previously described [27]. After 22 h, the cells were washed twice with PBS and lysed in 1x Reporter Lysis Buffer (Promega). A total of 50 µl of cell lysate was mixed with 50 µl of luciferase substrate (Promega) and light emission measured by Polarstar Optima (BMG Labtech, Ortenberg/Germany) luminometer. To assess Smad3 activation NRK52-E cells were grown in an 8-well chamber slide and treated with 12 µM Surfen before addition of pre activated Rh-TG2 as described above. After fixation in (3% PFA) and permeabilisation (0.1% (w/v) Triton X100), active Smad3 was detected by rabbit anti-Smad3(pSer425) polyclonal antibody (Sigma) followed by donkey anti rabbit Alexa 488. Total Smad3 was detected by rabbit anti-Smad3 polyclonal antibody (Cell Signalling) followed by donkey anti rabbit Alexa 488. Active Smad 3 was quantified by ImageJ intensity analysis.

Immunostaining of TG2 with Sdc1, HS or Versican in kidney cryosections

Cryostat sections (14 µm-thick) were processed and analysed as described in the main Methods for the co-staining of Sdc4 and TG2. The following antibodies were used: for Sdc1 and TG2 dual staining, mouse monoclonal anti-Sdc1 antibody (B-A38, Abcam) diluted 1:100 and rabbit polyclonal anti-TG2 antibody (ab421, Abcam), followed by, respectively, goat anti-mouse IgG DyLight 594 and donkey anti-rabbit IgG Alexa Flour 488; for HS and TG2 dual staining, mouse monoclonal IgM anti-HS antibody (10E4) diluted 1:50 and rabbit polyclonal anti-TG2 antibody (ab421, Abcam,) diluted 1:50, followed by, respectively, goat anti mouse

IgM-FITC and donkey anti-rabbit IgG Alexa Flour 568; for versican and TG2 dual staining rabbit monoclonal anti-versican antibody (EPR12277, Abcam) diluted 1:50 and mouse monoclonal anti-TG2 antibody (IA12, produced in Sheffield University) diluted 1:100, followed by, respectively, donkey anti-rabbit IgG Alexa Flour 488 and goat anti-mouse IgG Dy Light 594.
